# Fruit powder spray formulations reduce advanced glycation end-product formation in charcoal-grilled pork: effects of application method and polyphenol–sugar balance

**DOI:** 10.1016/j.crfs.2026.101592

**Published:** 2026-10-08

**Authors:** Mei-Ling Li, Shang-Ming Huang, Pei-Yu Wu, Ching-Wei Huang, Wei-Lun Hung, Kuo-Chiang Hsu

**Affiliations:** a Department of Nutrition, China Medical University, No.100, Sec.1, Jingmao Rd., Beitun Dist., Taichung City, 406040, Taiwan; b School of Food Safety, Taipei Medical University, No.250, Wuxing St., Xinyi Dist., Taipei City, 110, Taiwan; c Department of Food Nutrition and Health Biotechnology, Asia University, 500 Lioufeng Rd., Wufeng, Taichung, 41354, Taiwan

**Keywords:** Advanced glycation end-products, Processing strategy, Spray application, Polyphenols, Methylglyoxal trapping

## Abstract

Advanced glycation end-products (AGEs) formed during charcoal grilling of meat pose food safety concerns, yet few studies have compared how different methods of applying protective ingredients affect AGE formation. This study compared three application methods (marinating, spraying, and mixing) for delivering freeze-dried fruit powders and evaluated their effects on Nε-carboxymethyl-lysine (CML) and Nε-carboxyethyl-lysine (CEL) in charcoal-grilled pork loin and belly. Among eight fruit powders screened, strawberry (SB) and kumquat (KQ) were selected on the basis of their antioxidant-to-sugar balance. Two-way ANOVA showed that application method accounted for 62% of the variance in AGE inhibition in pork loin, compared with 13% for fruit type, and spraying maintained high inhibition across both lean and high-fat cuts. Soy sauce-free spray formulations reduced total AGEs by 83% (SB) and 86% (KQ) while maintaining low lipid oxidation, consumer acceptance above the neutral threshold, and stable performance during 90 days of accelerated storage at 45 °C. Molecular docking and methylglyoxal-trapping assays indicated that the fruit polyphenols may inhibit AGE formation through three complementary pathways: competitive binding at glycation-susceptible protein sites, direct scavenging of reactive carbonyl intermediates, and suppression of lipid oxidation. These findings show that how fruit powder is applied to meat can be as important as which fruit powder is used.

## Highlights

• Fruit powder sprays reduced glycation product formation in grilled pork by 83–86%.
• Application method explained more variation than fruit powder type.
• Antioxidant-to-sugar ratio alone did not fully predict inhibition performance.
• Polyphenol structure influenced carbonyl trapping and predicted protein binding.
• Soy sauce-free spray formulations maintained quality over 90 days of storage.

## Graphical abstract

**Figure:**
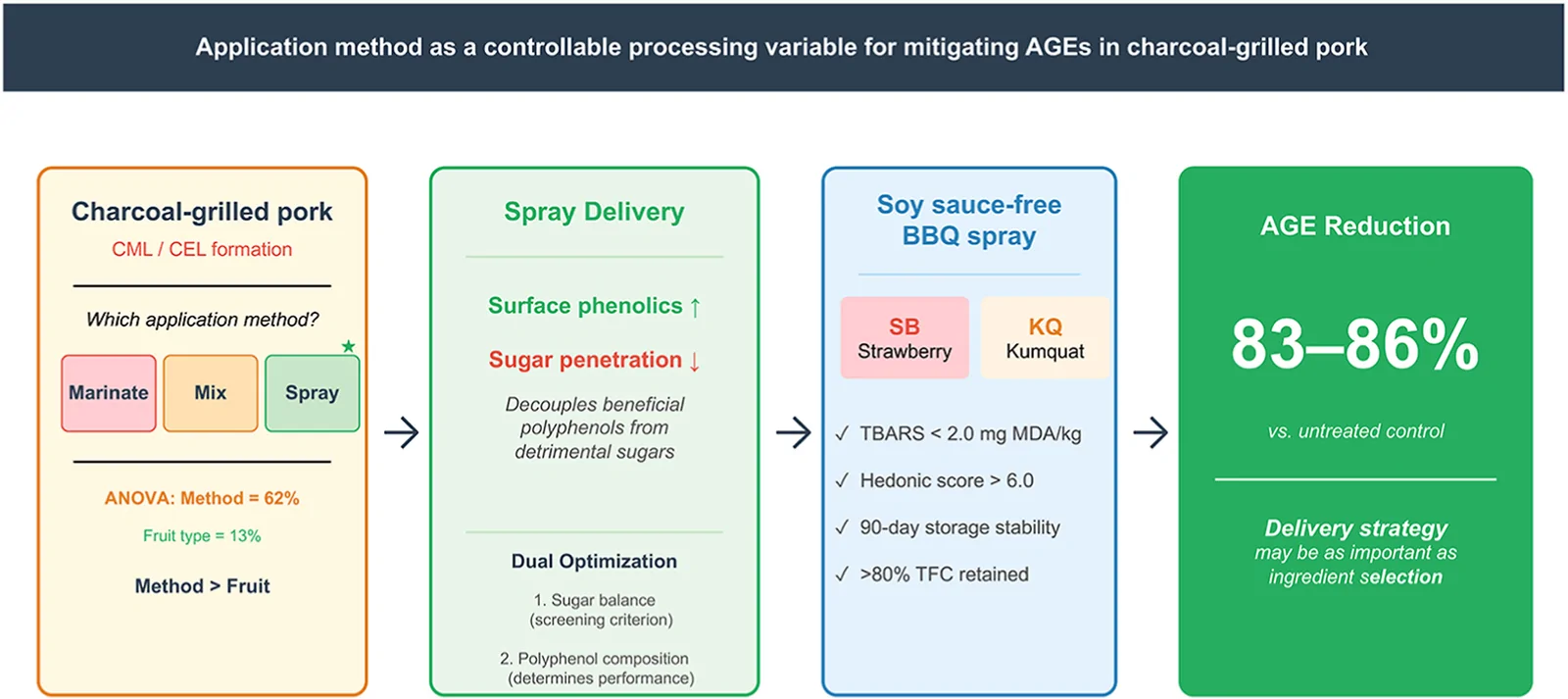

## 1 Introduction

Advanced glycation end-products (AGEs) are a structurally diverse class of compounds formed when reducing sugars react non-enzymatically with amino groups in proteins during thermal processing. Among the many AGE structures identified in foods, Nε-carboxymethyl-lysine (CML) and Nε-carboxyethyl-lysine (CEL) are the most widely used markers because of their abundance, chemical stability, and suitability for LC–MS/MS quantification (Scheijen et al., 2016; Troise et al., 2015). Dietary AGE intake has attracted growing attention because absorbed AGEs and their downstream signaling through the receptor for AGEs (RAGE) have been associated with chronic oxidative stress and low-grade inflammation (Nowotny et al., 2018; Vistoli et al., 2013). Charcoal grilling is of particular concern: direct flame contact and high surface temperatures markedly accelerate CML and CEL formation compared with other cooking methods (Eggen and Glomb, 2021). In Taiwan, pork accounts for 32.8% of total meat intake, and charcoal-grilled pork is ubiquitous in household and street-food settings. Identifying controllable processing variables that suppress AGE formation without compromising product quality has therefore become an important challenge in meat processing. Current approaches to AGE mitigation in meat systems include ingredient reformulation, marination with antioxidant-rich solutions, and adjustment of cooking parameters such as temperature and time (Uribarri et al., 2010; Poulsen et al., 2013). However, most of these strategies have been evaluated in model systems or under controlled oven-cooking conditions; their efficacy and feasibility under real charcoal-grilling conditions, where temperature control is inherently limited, remain less well characterized.

Natural polyphenol-rich materials have been widely explored as AGE inhibitors through mechanisms including free-radical scavenging, transition-metal chelation, and direct interception of reactive α-dicarbonyl intermediates such as methylglyoxal (MGO) and glyoxal (GO) (Khan et al., 2020; Peng et al., 2011). However, nearly all studies have focused on selecting or optimizing the active ingredient, whereas the method used to apply the protective agent to the meat surface has received little attention. Freeze-dried fruit powders are a particularly useful model system for investigating application method because they retain diverse phenolic profiles in a dry, formulation-compatible format while enabling standardized dosing (Kittibunchakul et al., 2023). In previous work, spray-applied fruit powder treatments reduced polycyclic aromatic hydrocarbons (PAHs) by up to 94.8% (Huang et al., 2024) and heterocyclic amines (HCAs) by up to 73.3% (Li et al., 2026) in the same charcoal-grilled pork system. However, PAH suppression proceeds mainly through pyrolytic pathways and HCA inhibition through Maillard precursor modulation, both of which are mechanistically distinct from the glycation and reactive carbonyl chemistry governing CML and CEL formation, so control of one hazard class does not predict control of the other. Consequently, whether the same application method can also mitigate AGE formation remains an open question.

Two critical gaps remain in translating fruit powder treatments for AGE reduction. First, most previous studies have focused on the selection or dose of the active ingredient, whereas the application method has rarely been treated as a controllable processing variable. Spray application enables controlled surface application without the prolonged immersion required for marination or the structural disruption of mixing, yet its effect on AGE mitigation relative to these conventional methods has not been systematically evaluated. Second, the choice of application method simultaneously determines the surface exposure of beneficial polyphenols and the amount of reducing sugars that reach the meat and promote AGE formation. Because fructose is 8–10-fold more reactive than glucose in Maillard chemistry (Schalkwijk et al., 2004), the net effect of a given fruit powder on AGE formation depends on the balance between these opposing constituents. The antioxidant-to-reducing-sugar ratio may therefore be a more informative selection criterion than absolute antioxidant capacity alone.

This study was based on the premise that effective AGE reduction requires attention to both ingredient composition and the method used to apply it, rather than optimizing either factor in isolation. Accordingly, this study aimed to (1) compare marinating, spraying, and mixing and quantify their relative contributions to AGE inhibition via factorial ANOVA; (2) develop and evaluate an antioxidant-to-(glucose + fructose) ratio as a formulation-screening criterion; (3) translate the optimized spray approach into sprayable BBQ sauces and assess technological feasibility (lipid-oxidation control, sensory acceptability, and storage stability); and (4) examine glycation-site blocking and carbonyl-scavenging mechanisms through molecular docking and MGO-trapping assays. In particular, this study sought to determine whether application method accounts for a larger proportion of AGE inhibition variance than ingredient selection.

## 2 Materials and methods

### 2.1 Materials and fruit powder preparation

Eight fresh fruits, guava (GV), strawberry (SB), lemon (LM), kumquat (KQ), papaya (PP), tankan (TK), passion fruit (PF), and banana (BN), were purchased from a local market in Taichung, Taiwan. All fruits were washed, trimmed as appropriate for each species, sliced, freeze-dried for 72 h, milled, and passed through a 40-mesh sieve. Powders were vacuum-sealed and stored at −20 °C until use. Detailed raw material specifications, analytical standards, and fruit-specific processing conditions are provided in Table S1.

### 2.2 Antioxidant characterization and sugar analysis

Fruit powder extracts prepared in 70% (v/v) ethanol were assayed for total phenolic content (TPC), total flavonoid content (TFC), ferric reducing antioxidant power (FRAP), and reducing power EC_50_ using established colorimetric methods (Table S2). The main soluble sugars (glucose, fructose, sucrose, lactose, and maltose) were quantified by HPLC with refractive index detection (Table S3). Eight powders were initially screened on both antioxidant and sugar profiles (Table S4); four (GV, SB, LM, KQ) were selected for subsequent experiments based on their consistently high antioxidant capacity relative to reducing-sugar content. TPC and TFC served as initial screening tools; compound-level characterization was subsequently performed by untargeted LC–MS/MS metabolomics (Section 2.5).

### 2.3 Pork treatment, application methods, and charcoal grilling

Commercial pork loin (*Longissimus dorsi*) and belly were used as model meats representing lean and high-fat matrices, respectively. A three-stage experimental program was employed: (i) dose–response testing with GV powder to identify the working concentration (Table S5); (ii) comparison of three application methods—marinating (4 °C, 2 h; absorption rate determined gravimetrically), spraying (calibrated atomizer, mass-matched to absorbed amount), and mixing (ground patties with equivalent dry-matter loading plus 20 g/kg NaCl)—at 48 g/L; and (iii) evaluation of sprayable BBQ sauces (Tables S6, S7, and S8). Two sprayable sauce bases were prepared: soy sauce-containing (BA) and soy sauce-free (BB). Each base was fortified with selected fruit powders, adjusted to pH 4.2–4.4, hot-filled into 150 mL spray bottles equipped with fine-mist nozzles, and applied to pork at 100 g/kg before grilling (Table S9). Charcoal grilling was conducted under standardized conditions (infrared-monitored charcoal bed temperature 500 ± 20 °C, Latin square spatial rotation, thermocouple-verified endpoint; see Table S7 for full protocol) until the geometric center reached 72 °C, as monitored by a thermocouple-equipped digital thermometer. Samples were cooled, homogenized, and analyzed within 24 h.

### 2.4 CML and CEL quantification

Grilled pork was defatted, reduced with sodium borohydride, acid-hydrolyzed (6 M HCl, 110 °C, 24 h), and spiked with CML-d4 as the internal standard. CML and CEL were quantified by LC–MS/MS using a hydrophilic interaction column in positive-electrospray multiple reaction monitoring (MRM) mode. Representative chromatograms are shown in Fig. S1; complete sample preparation procedures, instrument parameters, and MRM transitions are detailed in Table S10 and S11.

### 2.5 Mechanistic analyses

Untargeted LC–MS/MS metabolomics was used to profile polyphenols in KQ and SB powders. Twenty-eight representative compounds were selected from an initial pool of 1020 candidates through a four-layer filtering strategy (Table S12). These 28 polyphenols, together with three AGE precursors (glucose, MGO, GO), were docked against four glycation-susceptible hotspots on porcine myosin (AlphaFold-predicted structure, UniProt Q9TV62; Jumper et al., 2021) using AutoDock Vina 1.2.5 (exhaustiveness = 128; Eberhardt et al., 2021). The ability of individual polyphenols to trap methylglyoxal (a reactive carbonyl intermediate in AGE formation) was measured using four compounds that differ in the number of hydroxyl groups on their B-ring. Docking and trapping conditions are summarized in Table S13.

### 2.6 TBARS, sensory evaluation, and storage stability

Lipid oxidation was evaluated by the TBARS assay (Table S6). Consumer acceptance (n = 50, aged 20–51 years) was assessed using a 9-point hedonic scale across ten sensory attributes following ethics approval from the Research Ethics Committee of China Medical University and Hospital (CMUH114-REC2-065; Table S14 and S15). Accelerated shelf-life testing was conducted at 45 ± 2 °C for 90 days according to the TFDA guideline (TFDA, 2022) for prepackaged foods (Table S16). Samples were monitored at 0, 30, 60, and 90 days for color (ΔE), TFC, aerobic plate count, AGE inhibition, and TBARS on freshly grilled pork. Sensory discrimination was assessed by triangle tests (ISO 4120:2016; n = 10; Table S16).

### 2.7 Statistical analysis

All experiments were performed in triplicate and results are expressed as mean ± standard deviation. Statistical analysis was conducted using SAS 9.4. Two-way ANOVA was used to evaluate the variance contributions of application method, fruit type, and their interaction for AGE inhibition. Duncan's multiple range test was used for post-hoc comparisons at *p* < 0.05. Pearson correlation analysis was used to evaluate relationships between antioxidant parameters, sugar contents, antioxidant-to-sugar ratios, and AGE inhibition rates across different application conditions. For triangle tests, significance was assessed according to ISO 4120:2016.

## 3 Results and discussion

### 3.1 Fruit powder selection based on phenolic–sugar balance

Eight fruit powders were initially screened using TPC, TFC, FRAP, and reducing power EC_50_ (Table S4). GV, SB, LM, and KQ consistently outperformed PP, TK, PF, and BN across all four indicators. Table 1 presents the antioxidant capacity, sugar composition, and derived antioxidant-to-glucose ratios of the four selected powders. GV exhibited the highest FRAP (104.88 mg FeSO_4_ equivalents/g) and TPC (13.92 mg GAE/g) but also the heaviest total sugar burden (55.2 g/100 g dry powder), yielding a TPC/(Glu + Fru) ratio of only 0.35. In contrast, KQ combined the highest TFC (29.87 mg QE/g) with comparatively low glucose (7.9 g/100 g), resulting in the most favorable TPC/(Glu + Fru) (0.87) and TFC/(Glu + Fru) (2.02) ratios. SB, despite its moderate antioxidant capacity (FRAP 70.10), yielded a TPC/(Glu + Fru) ratio (0.35) identical to that of GV, owing to its comparably high reducing-sugar load (glucose 16.2, fructose 18.8 g/100 g). As demonstrated in Sections 3.2, 3.5, SB nevertheless achieved strong AGE inhibition through polyphenol-composition-dependent mechanisms not captured by the ratio alone. LM exhibited the highest TPC/(Glu + Fru) ratio (1.20) and TFC/(Glu + Fru) ratio (2.25) among all four powders, reflecting its exceptionally low reducing-sugar content (fructose 1.6 g/100 g) combined with flavanone-rich citrus peel inclusion. The reducing power EC_50_ values corroborated the FRAP hierarchy: GV (4.35 mg/mL) ≈ SB (4.93 mg/mL) > LM (5.56 mg/mL) ≫ KQ (12.20 mg/mL). Notably, KQ's high TFC but relatively low FRAP may reflect the structural characteristics of citrus flavanones, whose glycosylated forms reduce electron-donating efficiency in FRAP-type assays but may contribute to AGE inhibition through alternative mechanisms such as carbonyl trapping (Rice-Evans et al., 1996; Wu and Yen, 2005). Indeed, citrus-based powders (KQ and LM) exhibited markedly higher TFC than non-citrus powders, a pattern attributable to peel inclusion and the associated enrichment of flavanones such as hesperidin and naringin. These observations suggest that antioxidant metrics designed for electron-transfer reactions (FRAP, DPPH) may systematically undervalue fruit powders whose bioactivity operates primarily through carbonyl-trapping or metal-chelation pathways.

**Table 1:** Antioxidant capacity, reducing-sugar composition, and antioxidant-to-(fructose + glucose) ratios of the four selected fruit powders.

**Fruit powder**	**FRAP** (mg FeSO_4_ equivalents/g)	**Reducing power EC_50_** (mg/mL)	**TFC** (mg QE/g)	**TPC** (mg GAE/g)	**Fructose** (g/100 g)	**Glucose** (g/100 g)	**Total sugar** (g/100 g)	**TPC/(Fru + Glu)** ratio	**TFC/(Fru + Glu)** ratio
Guava (GV)	104.88 ± 1.62^a^	4.35 ± 0.07^a^	4.57 ± 0.40^de^	13.92 ± 0.40^a^	23.4 ± 2.26^a^	16.1 ± 1.32^a^	55.2 ± 4.38^a^	0.35	0.12
Strawberry (SB)	70.10 ± 2.80^b^	4.93 ± 0.06^ab^	9.74 ± 0.31^c^	12.32 ± 0.47^b^	18.8 ± 1.43^b^	16.2 ± 1.78^a^	38.9 ± 3.31^b^	0.35	0.28
Lemon (LM)	57.35 ± 8.49^c^	5.56 ± 0.06^ab^	22.90 ± 3.53^b^	12.25 ± 1.35^b^	1.6 ± 0.48^d^	8.6 ± 0.54^b^	13.0 ± 1.24^d^	1.20	2.25
Kumquat (KQ)	35.42 ± 4.81^d^	12.20 ± 0.43^c^	29.87 ± 4.51^a^	12.93 ± 0.29^ab^	6.9 ± 1.06^c^	7.9 ± 0.99^b^	21.9 ± 1.77^c^	0.87	2.02

FRAP, ferric reducing antioxidant power (mg FeSO_4_ equivalents/g); reducing power EC_50_, effective concentration giving an absorbance of 0.5; TFC, total flavonoid content (mg quercetin equivalents/g); TPC, total phenolic content (mg gallic acid equivalents/g).

Sugars are expressed as g/100 g dry powder; lactose and maltose were not detected in any sample.

TPC/(Fru + Glu) and TFC/(Fru + Glu) ratios were calculated from mean values as an index of antioxidant capacity relative to the combined fructose + glucose burden. Because fructose is 8–10-fold more reactive than glucose in the Maillard reaction, both reducing sugars were included in the denominator. No statistical comparison was applied to the derived ratios.

Values are mean ± SD (n = 3). For the antioxidant indices (FRAP, EC_50_, TFC, TPC), different superscript letters denote significant differences across the full eight-fruit screening (*p* < 0.05, one-way ANOVA followed by Duncan's multiple range test; see Supplementary Table S4). For the sugar variables, letters denote significant differences among the four selected powders. GV, guava; SB, strawberry; LM, lemon; KQ, kumquat.

This dual characterization is critical because fruit powders simultaneously deliver antiglycation polyphenols and reducing sugars that promote AGE formation to the meat surface. Fructose, 8–10-fold more reactive than glucose in the Maillard reaction (Schalkwijk et al., 2004), was the dominant monosaccharide in GV (23.4 g/100 g) and SB (18.8 g/100 g). Previous work has demonstrated that adding 10 g/kg glucose to pork increases the CML and CEL formation rate constants by 3.8- and 4.0-fold, respectively (Sun et al., 2021), highlighting the significant AGE-promoting potential of sugar-rich fruit ingredients. The antioxidant-to-reducing-sugar ratio therefore captures the net formulation potential more accurately than any single antioxidant metric. It should be noted, however, that the ratio serves as a necessary screening threshold rather than a sufficient predictor of AGE inhibition, because polyphenol composition and pathway-specific activity (Section 3.5) modulate the outcome independently of the sugar balance. This ratio-based approach differs from conventional screening strategies that rank fruit extracts solely by total polyphenol content or radical-scavenging capacity, and was adopted here as the primary selection criterion for subsequent meat experiments. In addition, the fructose-to-glucose ratio varied considerably among powders (GV 1.45, SB 1.16, KQ 0.87, LM 0.19), which may further modulate glycation kinetics given the higher Maillard reactivity of fructose.

### 3.2 Spray application improves AGE mitigation across pork matrices

The working concentration of 48 g/L was established by dose–response testing, which showed a plateau in AGE inhibition at ≥48 g/L (Table S5). Fig. 1 presents the AGE inhibition rates for all four fruit powders applied at 48 g/L via marinating, spraying, and mixing in pork loin and belly, together with the two-way ANOVA variance decomposition for each meat type. In pork loin, inhibition ranged from 23.2% (GV, spraying) to 73.0% (SB, mixing), and the application method was the dominant factor, accounting for 62% of total variance compared with 13% for fruit type and 17% for the method × fruit interaction. These variance components indicate that application method contributes more strongly to AGE inhibition than fruit selection in lean pork, demonstrating that processing variables can outweigh ingredient identity under appropriate matrix conditions. The mixing method, which ensures homogeneous distribution of fruit powder throughout the meat matrix, showed competitive or superior performance in loin (SB mixing: 73.0%; KQ mixing: 66.8%), consistent with enhanced contact between phenolic compounds and protein-lipid oxidation sites. However, mixing fundamentally alters the product form from intact cuts to restructured products, limiting its applicability in many commercial and consumer contexts.

**Fig. 1:**
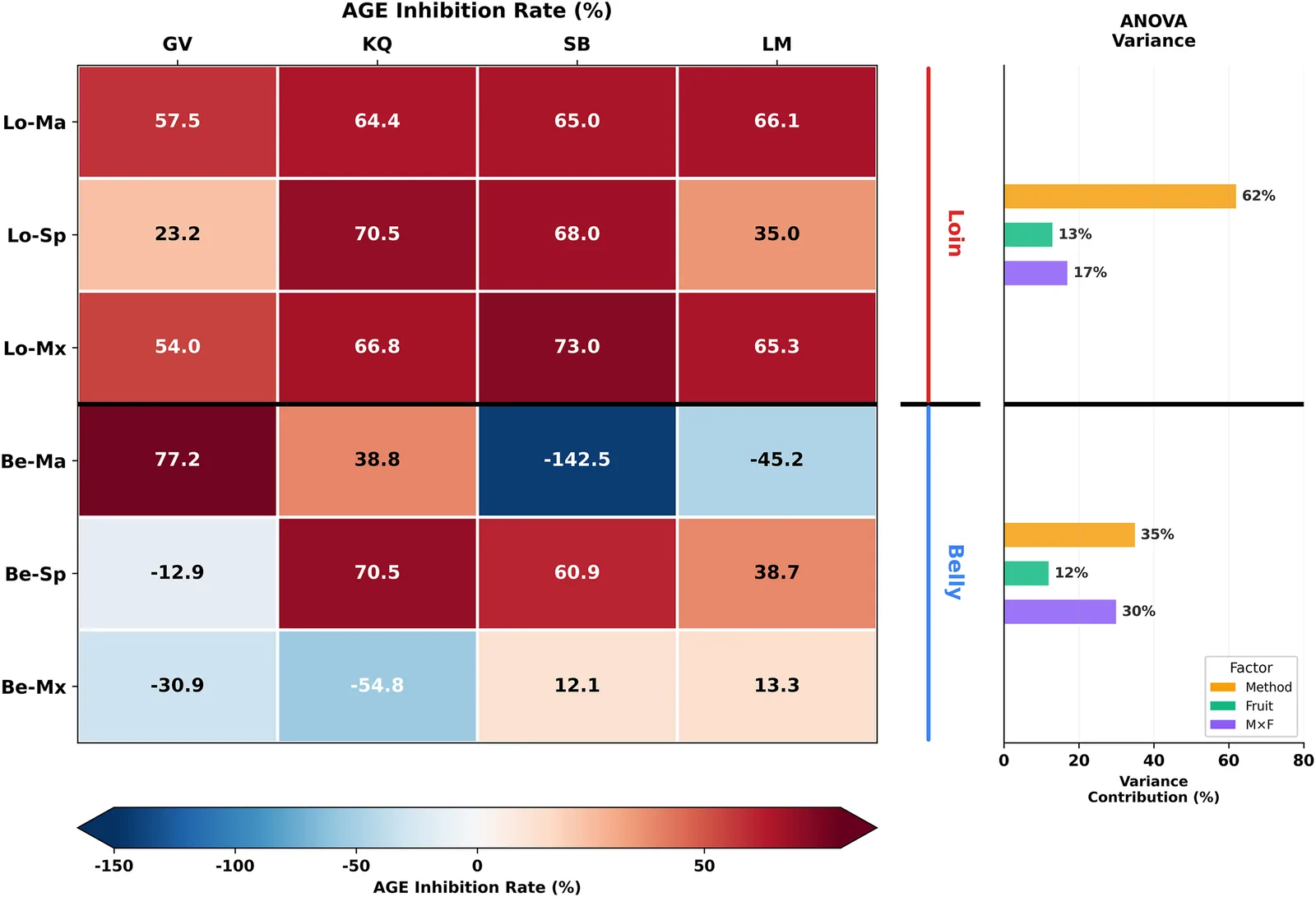
Effects of fruit powder type and application method on AGE inhibition (%) in charcoal-grilled pork loin (Lo) and belly (Be). Application methods: Ma, marinating; Sp, spraying; Mx, mixing. Right panel: two-way ANOVA variance contribution (%) of application method (orange), fruit type (green), and method × fruit interaction (purple). GV, guava; KQ, kumquat; SB, strawberry; LM, lemon.

The variance structure shifted markedly in pork belly. The method contribution fell to 35% while the method × fruit interaction rose to 30%, indicating that the high-fat matrix fundamentally alters the dynamics of AGE formation and inhibition. Marinating with SB in pork belly produced a paradoxical inhibition rate of −142.5%, likely because prolonged immersion allowed the high fructose and glucose loads of SB (18.8 and 16.2 g/100 g, respectively) to penetrate the fat-rich intramuscular space and overwhelm the protective effect of the co-delivered polyphenols. In contrast, spraying—which applies the formulation directly to the meat surface without the prolonged immersion used for marination—maintained >60% inhibition for both KQ (70.5%) and SB (60.9%) in belly. GV, despite having the highest FRAP among all powders, achieved only −12.9% inhibition (i.e., net promotion of AGE formation) when sprayed onto belly, further demonstrating that antioxidant capacity alone is insufficient to predict performance in fat-rich matrices. Although individual high-performing combinations were observed (e.g., GV belly-marinating 77.2%, SB loin-mixing 73.0%), spraying was selected as the preferred application method because it provided the most consistent inhibition across both lean and fatty matrices without requiring prolonged immersion or structural disruption of the meat. These observations indicate that spray application can retain the beneficial effects of fruit-derived polyphenols while limiting the adverse consequences associated with prolonged exposure to reducing-sugar-rich formulations during marination.

Pearson correlation analysis further supported the importance of the antioxidant-to-sugar balance. The TPC/(Glu + Fru) ratio (r = 0.61, *p* < 0.05) and TFC/(Glu + Fru) ratio (r = 0.73, *p* < 0.01) positively predicted AGE inhibition in the belly-marinating condition, whereas FRAP alone showed a significant negative correlation with inhibition in the loin-spraying condition (r = −0.69, *p* < 0.05) (Fig. S2). These results indicate that conventional antioxidant metrics can be misleading when applied to fruit powder treatments in meat, and that the phenolic-to-sugar ratio is a more informative formulation parameter than absolute antioxidant capacity, though its moderate correlation strength (r = 0.61–0.73) indicates that additional factors—notably polyphenol composition—co-determine the inhibition outcome (see Section 3.5). On the basis of these findings, spraying was selected as the application method and SB and KQ as the lead powders for BBQ sauce formulation. It is worth emphasizing that the residual (error) variance in the ANOVA was notably small in both loin (8%) and belly (23%), indicating that the three-factor model (method, fruit, and their interaction) captured the majority of systematic variation in AGE inhibition. This high explanatory power supports the use of a factorial design as a rational framework for optimizing fruit-powder-treated AGE mitigation strategies, rather than relying on single-variable screening approaches. The substantial interaction term in belly (30%) further underscores that the optimal fruit–method combination cannot be predicted from main effects alone and must be determined empirically for each meat matrix.

### 3.3 Soy sauce-free spray formulations maximize CML and CEL reduction

Two sprayable sauce bases were evaluated: soy sauce-containing (BA) and soy sauce-free (BB) (Table S9). Fig. 2 presents the CML and CEL contents and inhibition rates for all sauce–powder combinations in loin and belly. The BA blank itself elevated total AGEs in pork belly by 58.2% over the untreated control (from 2.02 to 3.19 μg/g), consistent with the Maillard-reactive free amino acids and reducing sugars generated during soy sauce fermentation (Diez-Simon et al., 2020). The BB blank, by contrast, reduced total AGEs by 27.4% in loin and 14.2% in belly relative to the control, demonstrating that the soy sauce-free matrix itself provides a less AGE-promoting background for polyphenol action. The AGE-promoting effect of soy sauce is consistent with its substantial content of free amino acids and reducing sugars, which serve as additional Maillard reaction substrates during grilling (Lin et al., 2023). This observation has direct formulation implications: eliminating soy sauce from the base not only removes a source of AGE-promoting precursors but also increases the effective concentration of fruit-powder-derived polyphenols relative to competing substrates. Interestingly, control pork belly (2.02 μg/g total AGEs) showed lower baseline AGE content than loin (2.59 μg/g), despite its higher fat content. This may be attributed to differences in protein accessibility: the abundant subcutaneous and intramuscular fat in belly physically shields protein surfaces from direct contact with reducing sugars and reactive carbonyls during grilling, resulting in lower Maillard reaction rates at equivalent surface temperatures.

**Fig. 2:**
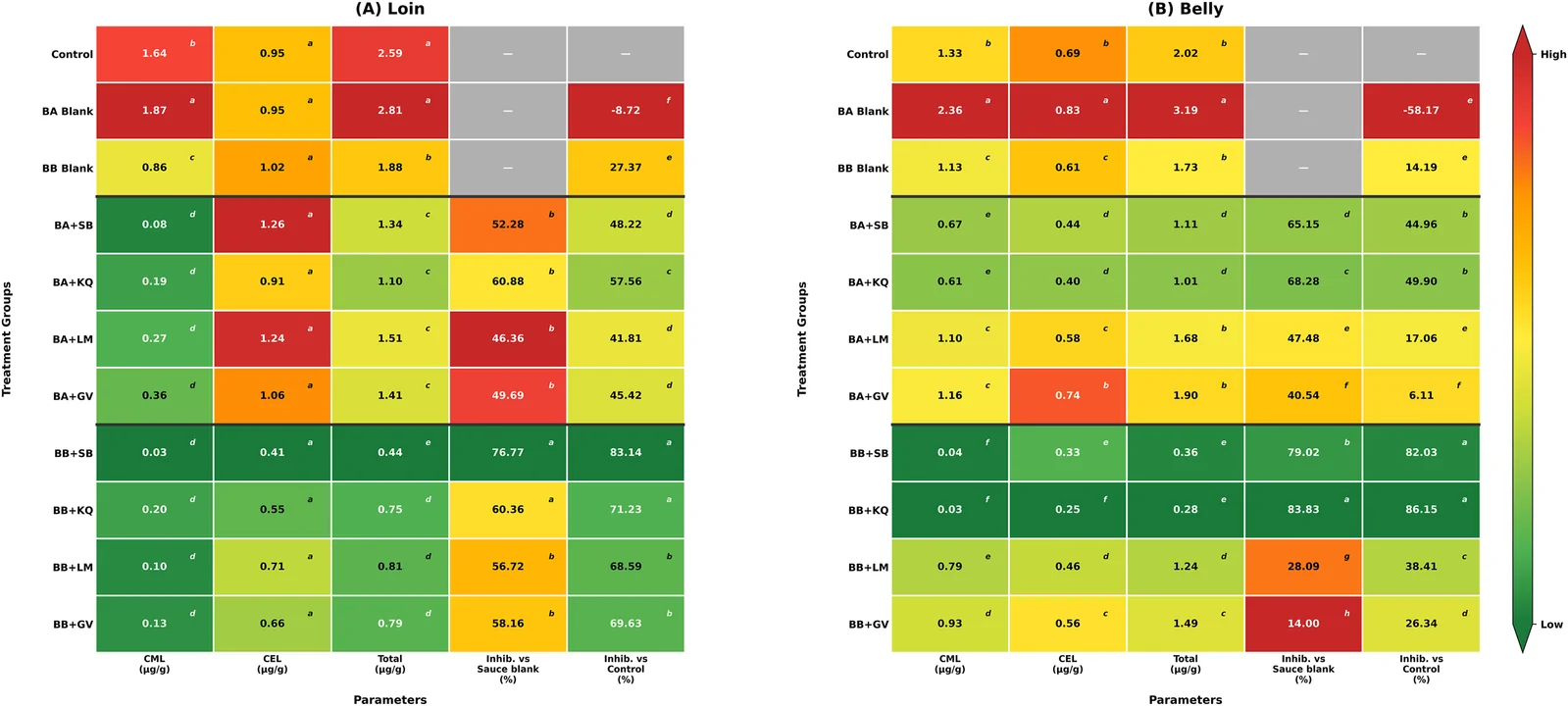
Comprehensive heatmap of CML and CEL contents and inhibition rates in grilled pork (A) loin and (B) belly treated with fruit powder-enriched BBQ sauces applied by spraying. BA, soy sauce-containing base; BB, soy sauce-free base. Different superscript letters within each column indicate significant differences (*p* < 0.05, Duncan's multiple range test). Gray cells: not applicable.

Among BB formulations, SB achieved the highest inhibition in loin (76.8% vs. sauce blank; 83.1% vs. control), reducing total AGEs from 2.59 to 0.44 μg/g, while KQ achieved the highest inhibition in belly (83.8% vs. sauce blank; 86.2% vs. control), with total AGEs falling from 2.02 to 0.28 μg/g. In the SB-BB loin treatment, CML was reduced from 1.64 to 0.03 μg/g (98.2% reduction) and CEL from 0.95 to 0.41 μg/g (56.8% reduction), indicating a preferential suppression of CML over CEL. A similar pattern was observed for KQ-BB in belly, where CML fell from 1.33 to 0.03 μg/g (97.7%) and CEL from 0.69 to 0.25 μg/g (63.8%). The greater susceptibility of CML to inhibition may indicate that the spray formulations more efficiently suppressed oxidative glycation routes leading to CML than MGO-associated pathways contributing to CEL, although direct precursor measurements would be needed to confirm this interpretation. The consistent superiority of BB over BA across all four fruit powders and both meat types indicates that the sauce base composition is not merely a background variable but an active modulator of net inhibitory performance. Notably, GV—despite possessing the highest FRAP among all powders—achieved only 6.1% inhibition versus control in the belly BA formulation, reinforcing the principle that high reducing-sugar loads can effectively negate antioxidant benefits even in a spray format. From a product-development perspective, the BB matrix offers a cleaner formulation window for maximizing AGE reduction while avoiding the confounding AGE-promoting contributions of fermented soy-based condiments. From a formulation perspective, these findings indicate that reducing the background load of Maillard-reactive precursors in the carrier system can substantially enhance the effectiveness of polyphenol-based approaches to reduce AGE formation. Among the four fruit powders tested, the BB formulation amplified the inhibitory advantage of powders with favorable antioxidant-to-sugar ratios (KQ and SB) while exposing the limitation of high-sugar powders (GV), further validating the ratio-based selection criterion established in Section 3.1.

It is also noteworthy that the AGE inhibition magnitudes achieved here (83–86% vs. control) compare favorably with previously reported natural antioxidant interventions in thermally processed meat systems (Khan et al., 2020; Nawaz et al., 2024). The combination of high efficacy with a simple spray application method represents a practical advance toward AGE control in grilled meat products.

### 3.4 Technological feasibility of selected SB and KQ formulations

For the selected SB-BB and KQ-BB formulations to be viable as practical processing aids, they must control lipid oxidation, satisfy consumer expectations, and remain functional during storage. Fig. 3A shows that both formulations significantly reduced TBARS values relative to the untreated control. SB-BB achieved 53.0% and 53.4% TBARS reduction in loin and belly, respectively, while KQ-BB achieved 32.3% and 37.6%. In absolute terms, SB-BB maintained TBARS at 0.205 ± 0.012 mg MDA/kg in loin and 0.389 ± 0.029 mg MDA/kg in belly, while KQ-BB maintained 0.295 ± 0.007 and 0.521 ± 0.033 mg MDA/kg, respectively. All treated samples remained well below the 2.0 mg MDA/kg level commonly associated with the onset of rancidity in meat products (Domínguez et al., 2019). The superior TBARS performance of SB over KQ is consistent with SB's higher FRAP value (70.10 vs. 35.42 mg FeSO_4_ equivalents/g) and its anthocyanin-rich phenolic profile, which provides stronger electron-donating capacity for scavenging lipid peroxyl radicals. Notably, the TBARS ranking (GV > SB > KQ) did not mirror the AGE inhibition ranking (KQ ≈ SB ≫ GV), confirming that lipid-oxidation control and glycation inhibition are governed by distinct mechanisms—a dissociation of practical importance for formulation design.

**Fig. 3:**
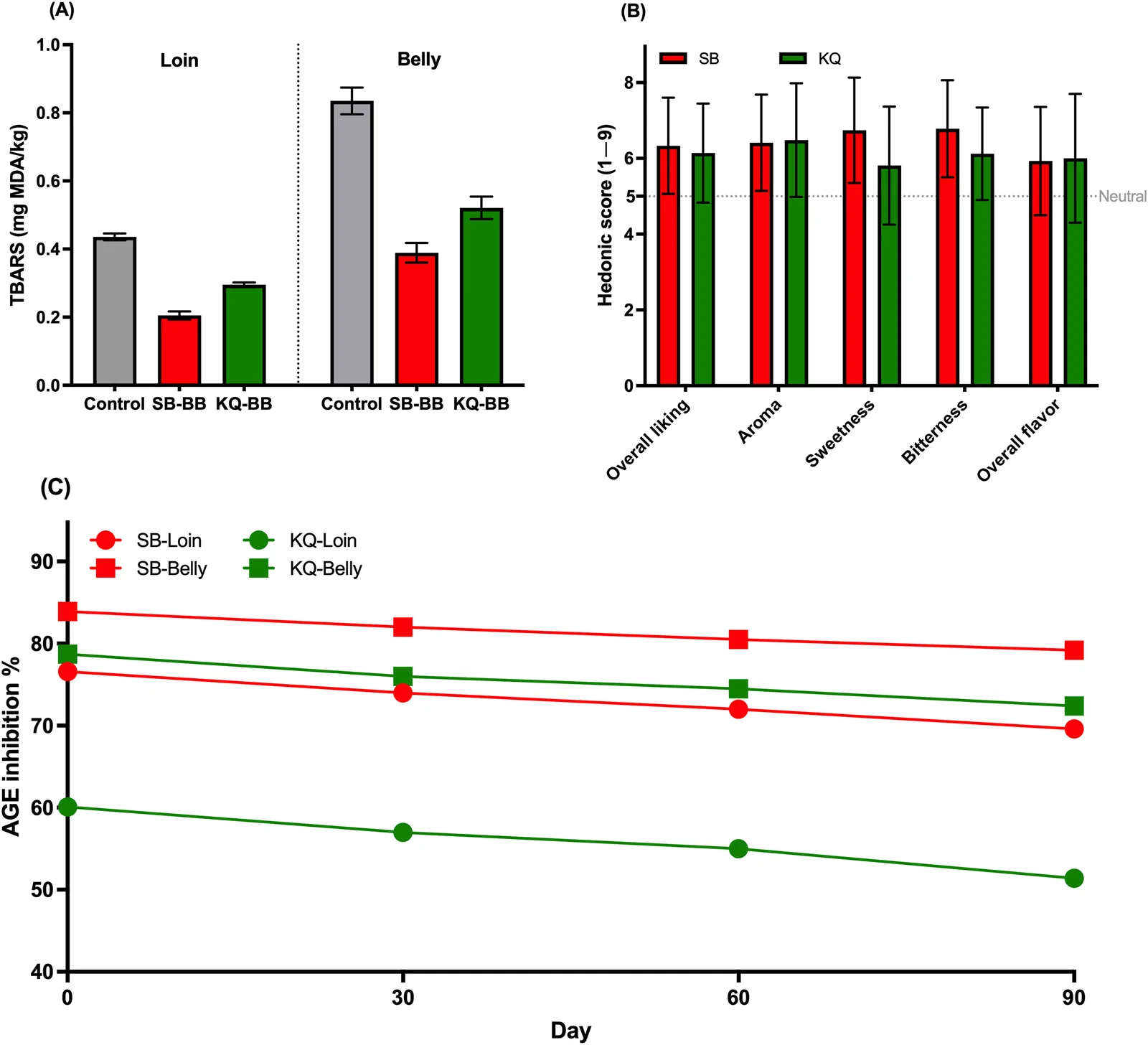
Technological feasibility of SB and KQ BB (soy sauce-free) formulations. (A) TBARS values of charcoal-grilled pork loin and belly treated with SB-BB and KQ-BB sauces versus untreated control. (B) Consumer acceptance scores (9-point hedonic scale, n = 50) for five key sensory attributes. Dashed line: neutral threshold (5.0). (C) Retention of AGE inhibition activity during accelerated storage at 45 °C for 90 days. Data: mean ± SD (n = 3). Significance vs. Day 0: *p < 0.05, **p < 0.01, ***p < 0.001.

Consumer acceptance testing (n = 50) confirmed that both SB (overall liking 6.33/9) and KQ (6.14/9) were well received (Fig. 3B). SB scored highest for sweetness acceptance (6.74) and bitterness acceptance (6.78), indicating that the anthocyanin-rich strawberry powder complemented the grilled pork flavor without imparting objectionable taste notes. KQ exhibited the best aroma (6.48) and aftertaste (6.19) scores, reflecting its appealing citrus fragrance derived from limonene and other terpene volatiles. Overall flavor scores were comparable between SB (5.93) and KQ (6.00), and both exceeded the neutral threshold. By contrast, LM received the lowest acceptance in the full four-powder screening (overall liking 5.44), primarily owing to pronounced bitterness (4.44) attributed to limonoids, and was therefore not advanced to storage testing. All sensory attributes for both SB and KQ formulations exceeded the neutral threshold of 5.0, confirming consumer acceptability (Table S17).

Accelerated storage (45 °C, 90 days) demonstrated sustained functional performance for both formulations (Fig. 3C; detailed storage parameters in Fig. S3). SB maintained AGE inhibition rates of 69.6–76.6% in loin and 79.2–83.9% in belly throughout the test period, while KQ showed 51.4–60.1% in loin and 72.4–78.7% in belly. Both sauces retained >80% of initial TFC and kept TBARS below 1.0 mg MDA/kg at all time points. Specifically, KQ retained 86% of its initial TFC (from 1.65 to 1.42 mg QE/g) and SB retained 81% (from 0.53 to 0.43 mg QE/g) after 90 days. The higher absolute TFC in KQ reflects its naturally greater flavanone content and suggests that KQ-based formulations may offer a longer functional window under extended storage. Aerobic plate counts remained within acceptable limits throughout the test period for both formulations, indicating satisfactory microbiological quality under the tested storage conditions. Triangle tests (n = 10) showed that overall flavor remained indistinguishable from the Day 0 reference throughout the entire 90 days (*p* > 0.05), although detectable changes in appearance and aroma emerged by Day 60–90, likely attributable to pigment degradation and terpene oxidation. Using a Q_10_ = 2 assumption, these accelerated results suggest the potential for extended ambient stability, although real-time storage validation remains necessary to confirm the projected shelf life. The superior overall stability of SB compared to KQ is likely attributable to the anthocyanin-rich phenolic profile of strawberry, which provides broader radical-scavenging coverage; KQ's volatiles, dominated by limonene and other monoterpenes, are more susceptible to oxidative degradation during storage, which may explain the earlier onset of detectable aroma changes. Nonetheless, both formulations met all functional benchmarks throughout the 90-day test, indicating that the proposed application method remains technologically feasible throughout storage without compromising its antiglycation performance.

### 3.5 Proposed mechanisms of AGE inhibition

While the factorial ANOVA identified application method as the dominant processing variable, the mechanistic basis underlying the different performances among fruit powders remained unresolved. To explore this, 28 polyphenols identified by untargeted metabolomics in KQ and SB (Table S18) were docked against four glycation-susceptible hotspots on porcine myosin. Of these, 27 (96.4%) exhibited stronger binding affinity than glucose (−5.10 kcal/mol), the most competitive AGE precursor, and all 28 surpassed both MGO (−2.74 kcal/mol) and GO (−2.19 kcal/mol) (Fig. S4; Table S18). On average, polyphenol binding was 2.0–2.8-fold stronger than AGE precursors across all four hotspots, consistent with a competitive site-blocking mechanism in which polyphenols may occupy Lys and Arg glycation sites on myosin that would otherwise react with reducing sugars or reactive carbonyl species. The top-ranked compounds included hesperidin (−8.98 kcal/mol at Hotspot 2), neohesperidin (−8.86 kcal/mol), and procyanidin B2 (−8.43 kcal/mol), all large glycosylated polyphenols with extensive hydrogen-bonding capacity. SB polyphenols exhibited a stronger average binding energy (−7.45 kcal/mol) than KQ polyphenols (−7.02 kcal/mol), both substantially exceeding all three AGE precursors.

MGO-trapping assays with four polyphenols with different numbers of free B-ring hydroxyl groups revealed a clear structure−activity relationship (Fig. 4; Table S19). Dihydromyricetin (pyrogallol B-ring, 3′,4′,5′-triOH) trapped 85.2 ± 2.1% of MGO, followed by catechin (catechol, 3′,4′-diOH; 65.0 ± 3.4%), pelargonidin-3-glucoside (mono 4′-OH; 33.9 ± 1.2%), and nobiletin (fully methoxylated; 2.1 ± 0.8%). Notably, nobiletin achieved moderate docking affinity (−6.48 kcal/mol) but near-zero trapping, whereas dihydromyricetin showed both strong binding (−7.76 kcal/mol) and the highest trapping capacity. This dissociation indicates that glycation-site blocking and carbonyl scavenging are governed by distinct structural features—molecular size and hydrogen-bonding groups for the former, free B-ring hydroxyl groups for the latter—and operate as complementary rather than redundant pathways. The stepwise increase in trapping capacity with increasing numbers of free B-ring hydroxyl groups is consistent with earlier reports identifying ortho-dihydroxyl (catechol) and 1,2,3-trihydroxyl (pyrogallol) configurations as structural features correlated with enhanced dicarbonyl scavenging in flavonoids (Wu and Yen, 2005).

**Fig. 4:**
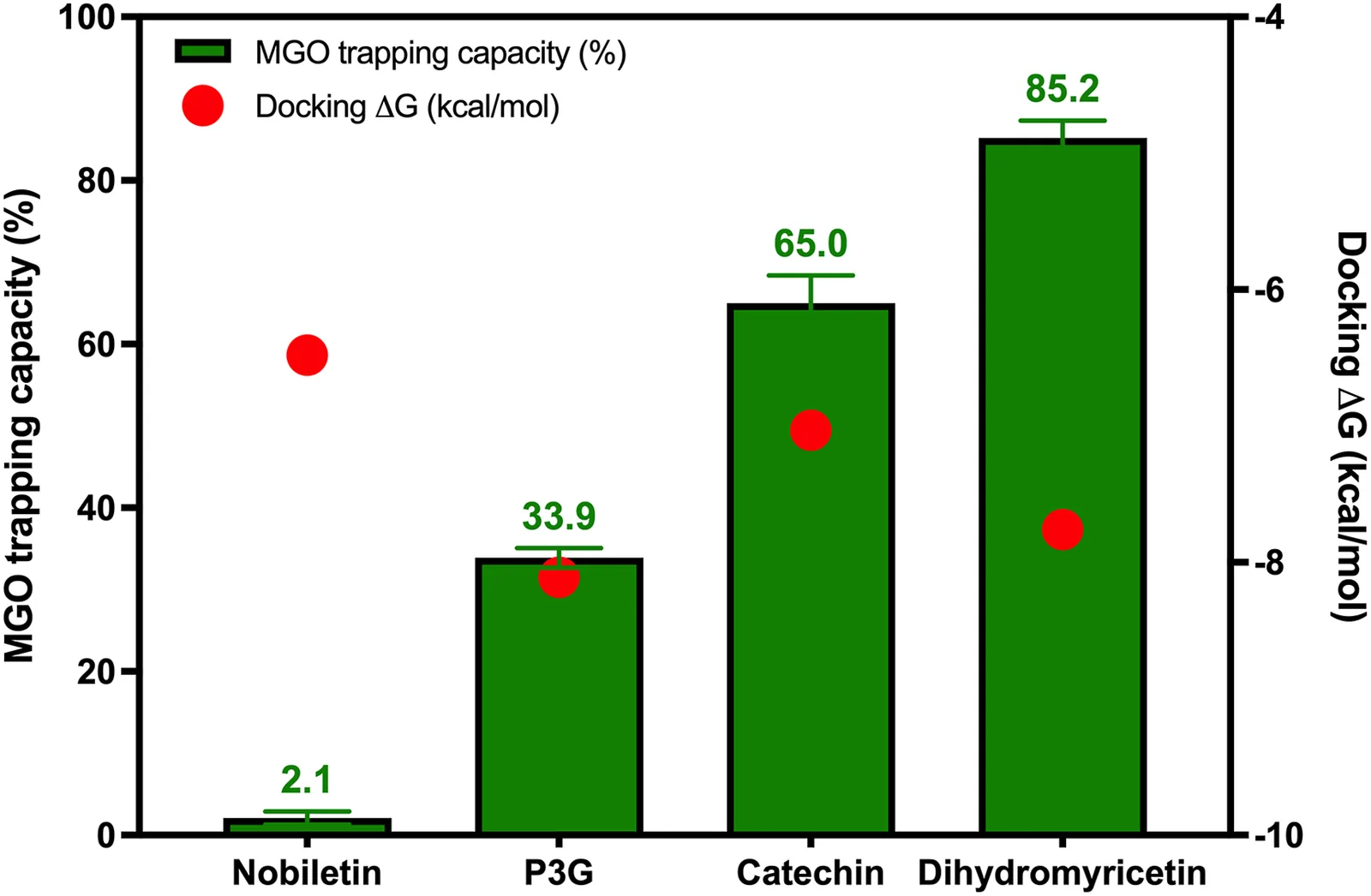
Relationship between B-ring hydroxylation pattern and MGO-trapping capacity of selected polyphenols. Bars: MGO trapping capacity (%); dots: best docking ΔG (kcal/mol) against porcine myosin glycation hotspots. Nobiletin (0 free B-ring OH), pelargonidin-3-glucoside (P3G, mono 4′-OH), catechin (3′,4′-diOH), dihydromyricetin (3′,4′,5′-triOH). Data: mean ± SD (n = 3).

Integrating these findings, a mechanistic model is proposed in Fig. 5 comprising three complementary pathways: (1) competitive binding of polyphenols at glycation-susceptible protein sites, blocking Maillard reaction initiation; (2) direct trapping of reactive α-dicarbonyl intermediates (MGO/GO), reducing the pool of glycation propagators; and (3) antioxidant-mediated suppression of lipid oxidation, decreasing the secondary reactive carbonyl species that feed back into AGE pathways (Zamora and Hidalgo, 2005). While the docking and trapping data do not constitute direct proof of these mechanisms in the grilled-meat matrix, they provide a plausible molecular framework consistent with the observed inhibition patterns and the complementary efficacy of SB and KQ. The stronger average binding affinity of SB polyphenols (−7.45 kcal/mol) compared to KQ (−7.02 kcal/mol) is consistent with the higher abundance of catechol- and pyrogallol-type flavonoids in strawberry that may engage both site-blocking and carbonyl-trapping pathways simultaneously. KQ's polyphenol profile, enriched in polymethoxyflavones such as nobiletin, may contribute to AGE inhibition primarily through antioxidant-mediated suppression of glycoxidation rather than direct carbonyl trapping. This mechanistic complementarity provides a rationale for the comparable overall AGE inhibition efficacy observed for both SB and KQ in the BB formulation, despite their distinct polyphenol compositions. These distinct profiles suggest that SB and KQ may provide complementary antiglycation functions under different meat-matrix and grilling conditions. The three-pathway model also provides a coherent explanation for the contrasting performance of individual fruit powders observed in Sections 3.1–3.3. KQ and SB achieved comparable overall AGE inhibition (86.2% and 83.1% vs. control) despite fundamentally different polyphenol profiles and a 2.5-fold difference in TPC/(Glu + Fru) ratio (KQ 0.87 vs. SB 0.35). KQ, enriched in polymethoxyflavones such as nobiletin, may act predominantly through Pathway 1 (competitive site-blocking; average docking ΔG −7.02 kcal/mol) and Pathway 3 (antioxidant-mediated suppression), with minimal contribution from Pathway 2 (nobiletin trapped only 2.1% of MGO). SB, by contrast, contains catechol- and pyrogallol-type flavonoids (catechin, dihydromyricetin) that showed the highest capacity for Pathway 2 (dihydromyricetin trapped 85.2% of MGO), which may help explain its strong AGE inhibition despite an unfavorable sugar balance. GV, which shares the same low ratio as SB (0.35), nevertheless showed poor performance in belly (−142.5% by marinating, −12.9% by spraying), further illustrating that the antioxidant-to-sugar ratio alone is insufficient to predict AGE inhibition. This per-fruit comparison suggests that the net inhibition outcome may be influenced by the compound-specific activation pattern across all three pathways, and that the antioxidant-to-sugar ratio may function as a useful but not sufficient screening threshold that should be complemented by compositional analysis of pathway-active polyphenols. Collectively, these findings indicate that successful antiglycation formulation requires two complementary levels of optimization. The antioxidant-to-sugar ratio provides an efficient first-stage screening criterion, whereas pathway-specific polyphenol composition determines the ultimate inhibitory performance. This dual-optimization concept explains why powders with similar antioxidant indices may exhibit markedly different activities in grilled meat systems. Future studies incorporating isotope-labeled precursors or protein-adduct mass spectrometry would be needed to validate these pathways under real grilling conditions.

**Fig. 5:**
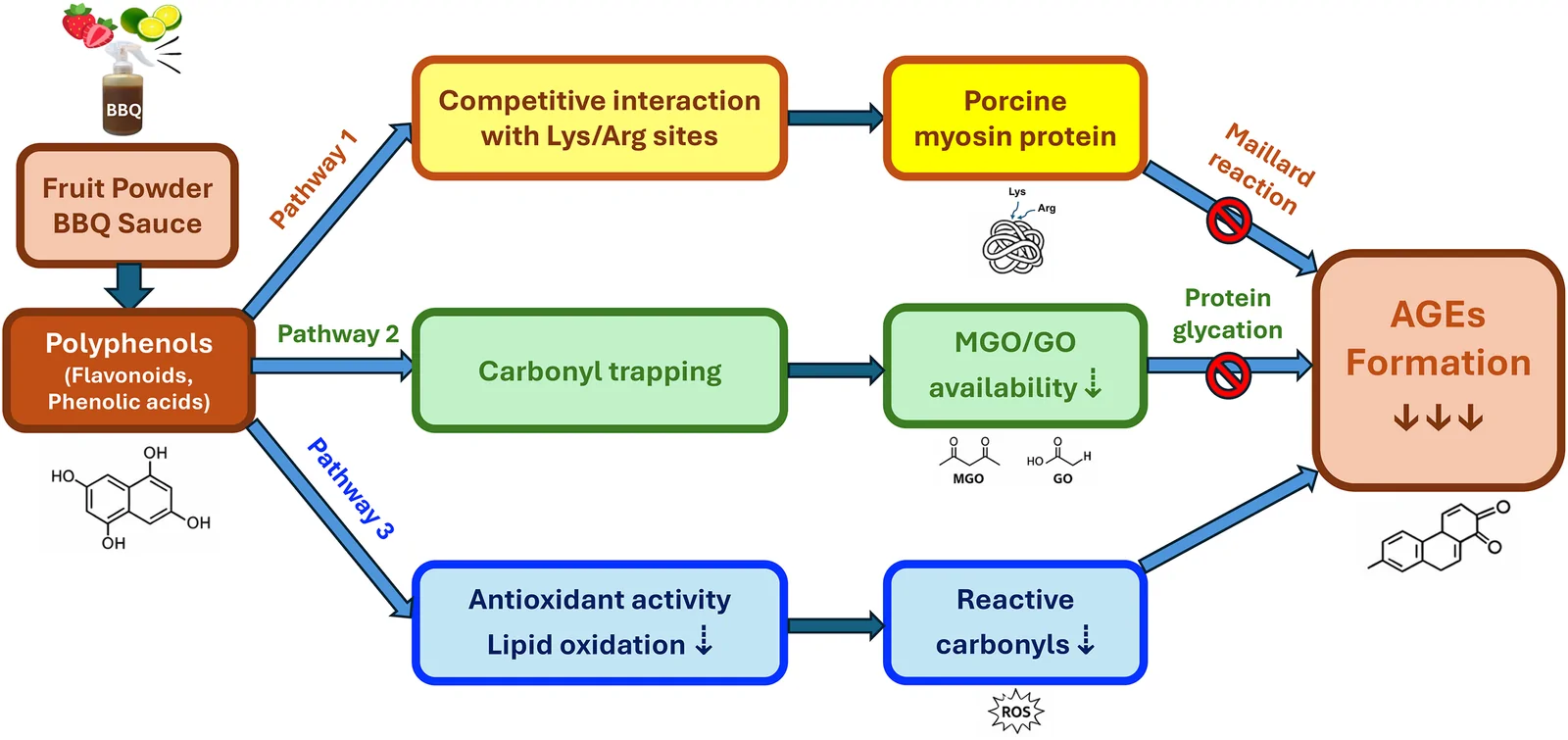
Proposed mechanisms of AGE inhibition by fruit powder BBQ sauce polyphenols. Pathway 1: competitive binding at glycation-susceptible Lys/Arg sites on porcine myosin (supported by molecular docking, Fig. S4). Pathway 2: direct trapping of reactive α-dicarbonyl intermediates MGO/GO (supported by MGO-trapping assay, Fig. 4). Pathway 3: antioxidant-mediated suppression of lipid oxidation and derived reactive carbonyls (supported by TBARS data, Fig. 3A).

## 4 Conclusions

This study demonstrates that spray application of fruit powder formulations is a feasible processing strategy for controlling CML and CEL formation in charcoal-grilled pork. The antioxidant-to-reducing-sugar ratio was identified as a more informative formulation-screening criterion than absolute antioxidant capacity, and application method explained a larger proportion of AGE inhibition variance than ingredient selection, particularly in high-fat matrices where marinating with high-sugar powders paradoxically promoted glycation. Soy sauce-free spray formulations with SB or KQ achieved 83–86% total AGE reduction while maintaining acceptable lipid-oxidation control (TBARS < 0.53 mg MDA/kg), consumer acceptance (hedonic scores > 6.1), and functional stability over 90 days at 45 °C (AGE inhibition retained > 50%, TFC retained > 80%). Molecular docking and MGO-trapping assays provided mechanistic support for complementary glycation-site blocking and carbonyl-scavenging pathways, with B-ring hydroxylation identified as a key structural determinant of trapping capacity. Several limitations should be acknowledged. First, the accelerated shelf-life test assumed linear Arrhenius behavior, and triangle tests detected changes in appearance and aroma by Day 60–90, so real-time storage validation is needed before full product shelf-life claims can be made. Second, the molecular docking and MGO-trapping analyses were performed in silico and in aqueous buffer, respectively; their quantitative relevance to the heterogeneous grilled-meat matrix remains to be established. Third, the correlation analyses were based on four fruit powders (n = 12 per condition), which constrains the generalizability of the ratio-based screening criterion to other plant-derived materials. Nonetheless, the convergence of inhibition data, structure–activity relationships, and functional stability results provides a coherent case for the practical viability of this approach. These results support spray-applied fruit powder formulations as a practical food-processing approach for mitigating heat-induced AGE formation in grilled pork, with potential applicability to other thermally processed meat products. Beyond the fruit powders examined here, these results suggest that the method used to apply polyphenol-rich ingredients to meat should be optimized alongside ingredient composition when developing practical approaches to reduce AGE formation in thermally processed foods. Further work should include real-time storage validation and pilot-scale production trials, and extension to other polyphenol-rich plant materials.

## CRediT authorship contribution statement

**Mei-Ling Li:** Methodology, Validation, Writing – original draft. **Shang-Ming Huang:** Resources, Formal analysis, Data curation. **Pei-Yu Wu:** Methodology, Data curation. **Ching-Wei Huang:** Investigation, Methodology, Data curation. **Wei-Lun Hung:** Supervision, Writing-review and editing. **Kuo-Chiang Hsu:** Conceptualization, Supervision, Writing – review & editing, Funding acquisition.

## Ethics declaration

Written informed consent to take part in the study and to publish the article has been obtained from all participants or their legal representatives. The privacy rights of participants have been observed.

This study was performed in compliance with relevant laws, regulatory frameworks and guidelines where the research took place. This study was conducted in accordance with ISO 4120:2021 (triangle test); ISO 8586:2023 (sensory assessors). This study was approved by the Research Ethics Committee of China Medical University and Hospital. (Approval No. CMUH114-REC2-065)

## Data availability statement

The source data supporting the findings of this study are available in Zenodo at https://doi.org/10.5281/zenodo.21292126 (Dataset S1).

## Declaration of generative AI and AI-assisted technologies in the writing process

During the preparation of this work, the authors used ChatGPT (OpenAI) and Claude (Anthropic) to improve language clarity, readability, and data visualization. These tools were not used to generate or interpret original data, produce scientific conclusions, or conduct peer review. All content was reviewed and edited by the authors, who take full responsibility for the integrity and accuracy of the work.

## Funding sources

This work was supported by the 10.13039/100020595National Science and Technology Council, Taiwan (grant number 10.13039/501100004663MOST 110-2320-B-039-042-MY3); the 10.13039/501100012544China Medical University, Taiwan (grant number CMU114-MF-38).

## Declaration of competing interest

The authors declare that they have no known competing financial interests or personal relationships that could have appeared to influence the work reported in this paper.

## References

[bib2] Diez-Simon C., Eichelsheim C., Mumm R., Hall R.D. (2020). Chemical and sensory characteristics of soy sauce: a review. J. Agric. Food Chem..

[bib1] Domínguez R., Pateiro M., Gagaoua M., Barba F.J., Zhang W., Lorenzo J.M. (2019). A comprehensive review on lipid oxidation in meat and meat products. Antioxidants.

[bib3] Eberhardt J., Santos-Martins D., Tillack A.F., Forli S. (2021). AutoDock vina 1.2.0: new docking methods, expanded force field, and python bindings. J. Chem. Inf. Model..

[bib4] Eggen M.D., Glomb M.A. (2021). Analysis of glyoxal- and methylglyoxal-derived advanced glycation end products during grilling of porcine meat. J. Agric. Food Chem..

[bib5] Huang S.M., Tung B.C., Hsieh C.H., Yang D.J., Huang C.W., Chang L.H., Hsu K.C. (2024). Inhibitory effects of fruit powders on the formation of polycyclic aromatic hydrocarbons in charcoal-grilled pork. Foods.

[bib6] International Organization for Standardization (ISO) (2016). Sensory analysis—Methodology—triangle Test.

[bib7] Jumper J., Evans R., Pritzel A., Green T., Figurnov M., Ronneberger O., Tunyasuvunakool K., Bates R., Žídek A., Potapenko A., Bridgland A., Meyer C., Kohl S.A.A., Ballard A.J., Cowie A., Romera-Paredes B., Nikolov S., Jain R., Adler J., Back T., Petersen S., Reiman D., Clancy E., Zielinski M., Steinegger M., Pacholska M., Berghammer T., Bodenstein S., Silver D., Vinyals O., Senior A.W., Kavukcuoglu K., Kohli P., Hassabis D. (2021). Highly accurate protein structure prediction with AlphaFold. Nature.

[bib8] Khan M., Liu H., Wang J., Sun B. (2020). Inhibitory effect of phenolic compounds and plant extracts on the formation of advance glycation end products: a comprehensive review. Food Res. Int..

[bib9] Kittibunchakul S., Temviriyanukul P., Chaikham P., Kemsawasd V. (2023). Effects of freeze drying and convective hot-air drying on predominant bioactive compounds, antioxidant potential and safe consumption of maoberry fruits. Lebensm. Wiss. Technol..

[bib10] Li M.L., Lin Y.S., Wu P.Y., Chang L.H., Huang S.M., Hsu K.C. (2026). Inhibitory effects of fruit powders on heterocyclic amines formation in charcoal-grilled pork: a comparative study of application methods and development of a functional barbecue sauce spray. Food Res. Int..

[bib11] Lin Y.Y., Huang S.F., Liao K.W., Ho C.T., Hung W.L. (2023). Quantitation of α-dicarbonyls, lysine- and arginine-derived advanced glycation end products, in commercial canned meat and seafood products. J. Agric. Food Chem..

[bib12] Nawaz A., Walayat N., Khalifa I., Harlina P.W., Irshad S., Qin Z., Luo X. (2024). Emerging challenges and efficacy of polyphenols-proteins interaction in maintaining the meat safety during thermal processing. Compr. Rev. Food Sci. Food Saf..

[bib13] Nowotny K., Schröter D., Schreiner M., Grune T. (2018). Dietary advanced glycation end products and their relevance for human health. Ageing Res. Rev..

[bib14] Peng X., Ma J., Chen F., Wang M. (2011). Naturally occurring inhibitors against the formation of advanced glycation end-products. Food Funct..

[bib15] Poulsen M.W., Hedegaard R.V., Andersen J.M., de Courten B., Bügel S., Nielsen J., Skibsted L.H., Dragsted L.O. (2013). Advanced glycation endproducts in food and their effects on health. Food Chem. Toxicol..

[bib16] Rice-Evans C.A., Miller N.J., Paganga G. (1996). Structure-antioxidant activity relationships of flavonoids and phenolic acids. Free Radic. Biol. Med..

[bib17] Schalkwijk C.G., Stehouwer C.D.A., van Hinsbergh V.W.M. (2004). Fructose-mediated non-enzymatic glycation: sweet coupling or bad modification. Diabetes Metabol. Res. Rev..

[bib18] Scheijen J.L.J.M., Clevers E., Engelen L., Dagnelie P., Brouns F., Stehouwer C.D.A., Schalkwijk C.G. (2016). Analysis of advanced glycation endproducts in selected food items by ultra-performance liquid chromatography tandem mass spectrometry: presentation of a dietary AGE database. Food Chem..

[bib19] Sun X., Li X., Tang J., Lai K., Rasco B.A., Huang Y. (2021). Formation of protein-bound N^ε^-carboxymethyllysine and N^ε^-carboxyethyllysine in ground pork during commercial sterilization as affected by the type and concentration of sugars. Food Chem..

[bib20] TFDA (2022).

[bib21] Troise A.D., Fiore A., Wiltafsky M., Fogliano V. (2015). Quantification of Nε-(2-Furoylmethyl)-L-lysine (furosine), Nε-(Carboxymethyl)-L-lysine (CML), Nε-(Carboxyethyl)-L-lysine (CEL) and total lysine through stable isotope dilution assay and tandem mass spectrometry. Food Chem..

[bib22] Uribarri J., Woodruff S., Goodman S., Cai W., Chen X., Pyzik R., Yong A., Striker G.E., Vlassara H. (2010). Advanced glycation end products in foods and a practical guide to their reduction in the diet. J. Am. Diet. Assoc..

[bib23] Vistoli G., De Maddis D., Cipak A., Zarkovic N., Carini M., Aldini G. (2013). Advanced glycoxidation and lipoxidation end products (AGEs and ALEs): an overview of their mechanisms of formation. Free Radic. Res..

[bib24] Wu C.H., Yen G.C. (2005). Inhibitory effect of naturally occurring flavonoids on the formation of advanced glycation endproducts. J. Agric. Food Chem..

[bib25] Zamora R., Hidalgo F.J. (2005). Coordinate contribution of lipid oxidation and Maillard reaction to the nonenzymatic food browning. Crit. Rev. Food Sci. Nutr..

