# Supplementary material for: Fruit powder spray formulations reduce advanced glycation end-product formation in charcoal-grilled pork: effects of application method and polyphenol–sugar balance
Source: Curr Res Food Sci. 2026 Oct 8;13:101592. doi: 10.1016/j.crfs.2026.101592 (PMC13677761; doi:10.1016/j.crfs.2026.101592)
Supplement: Supplementary file 1 [file mmc2.docx]

**Supplementary Material**

*“Application method as a controllable processing variable for mitigating advanced glycation end-products in charcoal-grilled pork: insights from fruit powder spray formulations”*

**Table S1.** Raw materials, standards, and fruit-powder preparation details.

| **Item** | **Description retained in supplement** |
| --- | --- |
| Fruit materials | Fresh guava (GV), lemon (LM), kumquat (KQ), and strawberry (SB) purchased from a local market in Taichung, Taiwan. |
| Trimming | SB: calyx and stem removed. GV, LM, and KQ: seeds removed and peel retained. |
| Slicing | All fruits sliced to approximately 4 mm thickness before drying. |
| Freeze-drying | FD12-4P-D freeze-dryer (Kesin Scientific Co., New Taipei, Taiwan), 72 h. |
| Milling and sieving | Ground and passed through a 40-mesh sieve. |
| Storage | Fruit powders sealed and stored at −20°C until use. |
| AGE standards | CML, CEL, and CML-d_4_ standards purchased from LGC Standards (Teddington, UK). |
| General reagents | All other chemicals and reagents were of analytical grade. |

**Table S2.** Fruit-powder extraction and antioxidant assay conditions.

| **Assay / step** | **Condition retained in supplement** | **Response unit** |
| --- | --- | --- |
| Extraction solvent | 1 g fruit powder + 10 mL of 70% (v/v) ethanol; vortex 2 min; ultrasonic extraction 30 min; centrifuge at 4,000 rpm for 20 min at 4°C. | Extract used for all antioxidant assays. |
| TPC | 50 μL extract + 450 μL water + 2.5 mL 0.2 N Folin-Ciocalteu reagent; after 8 min add 2 mL saturated Na_2_CO_3_; stand 30 min in the dark; read at 765 nm; gallic acid 0-500 μg/mL. | mg GAE/g  (Molole et al., 2022) |
| TFC | 1 mL extract + 4 mL water + 300 μL 50 g/L NaNO_2_ (5 min) + 300 μL 100 g/L AlCl_3_ (1 min) + 2 mL 1 M NaOH; read at 415 nm; quercetin 0-200 μg/mL. | mg QE/g  (Moo-Huchin et al., 2014) |
| FRAP | 50 μL extract + 150 μL FRAP reagent; stand 5 min; read at 593 nm. | mg FeSO_4_ equivalents/g  Benzie & Strain (1996) |
| Reducing power | 2.5 mL extract + 2.5 mL 0.2 M phosphate buffer pH 6.6 + 2.5 mL 10 g/L potassium ferricyanide (50°C, 20 min) + 2.5 mL 100 g/L trichloroacetic acid; 5 mL supernatant + 5 mL distilled water + 1 mL 1 g/L ferric chloride; read at 700 nm; EC_50_ was defined as the concentration achieving absorbance of 0.5. | mg/mL  (Ferreira et al., 2007) |

**Table S3.** HPLC-RI conditions for sugar composition analysis.

| **Parameter** | **Condition** |
| --- | --- |
| Instrument | HPLC with refractive index detection. |
| Column | Lichrospher NH_2_, 5 μm, 4.0 × 250 mm. |
| Mobile phase | Acetonitrile:water = 82:18 (v/v). |
| Flow rate | 1.0 mL/min. |
| Internal standard | Xylose, 100 g/L. |
| Sample preparation | Sample mixed with internal standard and 50% (v/v) ethanol, diluted to 100 mL, centrifuged at 10,000 rpm for 15 min, and filtered through a 0.45 μm membrane. |
| Analytes | Glucose, fructose, sucrose, lactose, and maltose. |

**Table S4.** Antioxidant activities, total flavonoid content, and total phenolic content of eight Taiwanese fruit powders.

| **Fruit powder** | **FRAP ^1^** | **Reducing power EC_50_ ^2^** | **TFC ^3^** | **TPC ^4^** |
| --- | --- | --- | --- | --- |
|  | (mg FeSO_4_ equivalents/g) | (mg/mL) | (mg QE/g) | (mg GAE/g) |
| Guava | 104.88 ± 1.62 ^a^ | 4.35 ± 0.07 ^a^ | 4.57 ± 0.40 ^de^ | 13.92 ± 0.40 ^a^ |
| Strawberry | 70.10 ± 2.80 ^b^ | 4.93 ± 0.06 ^ab^ | 9.74 ± 0.31 ^c^ | 12.32 ± 0.47 ^b^ |
| Lemon | 57.35 ± 8.49 ^c^ | 5.56 ± 0.06 ^ab^ | 22.90 ± 3.53 ^b^ | 12.25 ± 1.35 ^b^ |
| Papaya | 37.61 ± 2.35 ^d^ | 6.83 ± 1.09 ^b^ | 1.00 ± 0.04 ^f^ | 6.39 ± 0.21 ^c^ |
| Kumquat | 35.42 ± 4.81 ^d^ | 12.20 ± 0.43 ^c^ | 29.87 ± 4.51 ^a^ | 12.93 ± 0.29 ^ab^ |
| Tankan | 12.03 ± 0.85 ^e^ | 23.98 ± 1.44 ^d^ | 6.48 ± 0.31 ^c^ | 5.29 ± 0.12 ^d^ |
| Passion fruit | 4.92 ± 0.57 ^f^ | 26.50 ± 0.27 ^e^ | 4.29 ± 0.32 ^def^ | 2.42 ± 0.67 ^e^ |
| Banana | 1.68 ± 0.09 ^f^ | 46.21 ± 2.80 ^f^ | 1.51 ± 0.07 ^ef^ | 1.18 ± 0.08 ^f^ |

^1^ FRAP: ferric reducing antioxidant power; FeSO_4_: ferrous sulfate equivalents.

^2^ Reducing power EC_50_: effective concentration at which the absorbance is 0.5.

^3^ TFC: total flavonoid content; QE: quercetin equivalents.

^4^ TPC: total phenolic content; GAE: gallic acid equivalents.

^a–f^ Values are expressed as mean ± SD (n = 3). Different superscript letters within the same column indicate significant differences (*p* < 0.05) as determined by one-way ANOVA followed by Duncan's multiple range test.

**Table S5.** Effect of GV powder aqueous solution on the formation of CML and CEL in charcoal-grilled pork loin marinated at 4 °C for 2 h.

| **GV powder**  **concentration (g/L)** | **CML**  **(μg/g)** | **CEL**  **(μg/g)** | **CML + CEL**  **(μg/g)** | **Inhibition**  **(%)** |
| --- | --- | --- | --- | --- |
| 0 (Water) | 34.91 ± 3.73 ^a^ | 16.08 ± 0.72 ^a^ | 50.99 ± 4.07 ^a^ | — |
| 12 | 29.48 ± 3.01 ^ab^ | 12.90 ± 2.29 ^ab^ | 42.39 ± 4.91 ^ab^ | 16.88 ± 9.63 ^b^ |
| 24 | 26.99 ± 5.33 ^b^ | 13.12 ± 2.59 ^ab^ | 40.11 ± 7.20 ^b^ | 21.34 ± 14.13 ^b^ |
| 36 | 22.52 ± 4.63 ^b^ | 13.21 ± 0.51 ^ab^ | 35.73 ± 4.77 ^b^ | 29.92 ± 9.35 ^b^ |
| 48 | 11.78 ± 2.47 ^c^ | 10.18 ± 3.00 ^bc^ | 21.96 ± 5.44 ^c^ | 56.94 ± 10.67 ^a^ |
| 60 | 9.04 ± 0.61 ^c^ | 8.03 ± 1.82 ^c^ | 17.07 ± 2.03 ^c^ | 66.53 ± 3.98 ^a^ |

GV, guava; CML, N^ε^-(carboxymethyl)lysine; CEL, N^ε^-(carboxyethyl)lysine. Values are expressed as mean ± standard deviation (n = 3). ^a–c^ Different superscript letters within the same column indicate significant differences (*p* < 0.05) as determined by one-way ANOVA followed by Duncan's multiple range test.

**Table S6.** Overview of experimental modules, sample groups, response variables, and replication.

| **Module** | **Purpose and treatments** | **Key response variables** | **Replication / notes** |
| --- | --- | --- | --- |
| Dose-response screening | GV powder at 0, 12, 24, 36, 48, and 60 g/L to identify the working concentration for subsequent studies. | CML and CEL inhibition. | Triplicate preparation. |
| Application-method comparison | Marinating, spraying, and mixing using selected fruit powders at 48 g/L or equivalent dry-matter input. | CML, CEL, TBARS. | Triplicate; pork loin and belly. |
| Fruit selection | Comparison among GV, SB, KQ, and LM based on antioxidant properties and antiglycation performance. | Antioxidant indices, sugar profile, AGE inhibition. | Used to select powders for sauce development. |
| Mechanistic studies | Untargeted metabolomics, molecular docking, and MGO-trapping assays using representative polyphenols. | Binding affinity, hotspot occupancy, MGO trapping. | Docking parameters in S13. |
| Sauce efficacy test | Soy sauce-containing base (BA) and soy sauce-free base (BB) fortified with selected fruit powders. | CML, CEL, TBARS, sensory acceptance. | Application by spraying at 100 g/kg. |
| Accelerated shelf-life study | Storage of sauce at 45 ± 2°C for 90 days, sampled at 0, 30, 60, and 90 d. | Color, TFC, APC, AGE inhibition, TBARS, triangle test. | Q10-based interpretation described in main text. |

**Table S7.** Pork treatment, sauce application, and charcoal-grilling conditions.

| **Category** | **Operational detail retained in supplement** |
| --- | --- |
| Pork cuts | Commercial pork loin (Longissimus dorsi) and belly from a local meat purveyor; stored at 4°C and processed within 24 h. |
| Freshness screening | Visual inspection for color and exudate, tactile assessment for firmness and elasticity, and olfactory screening for off-odors. |
| Portion standardization | Thickness 0.5 ± 0.1 cm; dimensions 11 ± 0.2 cm × 7 ± 0.2 cm; weight 20 ± 2 g. |
| Marinating | 48 g/L fruit powder solution; liquid-to-solid ratio 1:0.8 (w/w); 4°C; 2 h; absorption rate calculated gravimetrically. |
| Spraying | 48 g/L solution delivered by calibrated atomizer at 0.2 ± 0.02 g per actuation; 5 sprays per side; total 2.0 g per portion. |
| Mixing | Pork ground through 5-mm plate; fruit powder added at marination-equivalent dry-matter level; 20 g/kg NaCl added; mixed until homogeneous (~60 s) and formed into 60-mm patties. |
| Sauce processing | BA (soy sauce-containing) and BB (soy sauce-free) bases; pH adjusted to 4.2–4.4 using white vinegar; hot filled at 93-95°C after passing through a 40-mesh sieve; packaged in 150-mL spray bottles with fine-mist nozzle. |
| Sauce application | Applied to pork at 100 g/kg sauce-to-meat ratio by spraying. |
| Charcoal quantity | Approximately 1.5 kg hardwood charcoal per session, arranged in a uniform monolayer and ignited with a propane torch. |
| Grill readiness | Grilling started when charcoal developed a complete white-gray ash layer. |
| Grill temperature | Charcoal bed monitored every 60 s by infrared thermometer; target 500 ± 20°C; grate-to-charcoal distance 110 mm. |
| Cooking procedure | Samples grilled on preheated stainless-steel grates and turned every 60 s; spatial position rotated according to a Latin square design. |
| Thermal endpoint | Core temperature 72°C verified using a Type K thermocouple (1.5 mm diameter; response time <5 s). |
| Typical cooking time | Loin 7.0 ± 0.5 min; belly 5.0 ± 0.3 min. |
| Post-cooking handling | Immediately cooled in an ice bath for 5 min, homogenized in a food processor (1,000 rpm, 60 s), and analyzed within 24 h. |
| Replication | All treatment combinations prepared in triplicate. |

**Table S8.** Effects of fruit powder (48 g/L) application methods on AGEs content (μg/g) in grilled pork.

| **Treatment** | **Marinating** | | |  | **Spraying** | | |  | **Mixing** | | |
| --- | --- | --- | --- | --- | --- | --- | --- | --- | --- | --- | --- |
|  | **CML** | **CEL** | **CML+CEL** |  | **CML** | **CEL** | **CML+CEL** |  | **CML** | **CEL** | **CML+CEL** |
| **Pork loin** |  |  |  |  |  |  |  |  |  |  |  |
| Control | 2.06 ± 0.53 ^a^ | 0.63 ± 0.08 ^a^ | 2.69 ± 0.55 ^a^ |  | 4.31 ± 0.53 ^a^ | 3.28 ± 0.84 ^a^ | 7.59 ± 1.37 ^a^ |  | 2.66 ± 0.29 ^a^ | 1.46 ± 0.24 ^a^ | 4.12 ± 0.52 ^a^ |
| SB | 0.45 ± 0.17 ^b^ | 0.50 ± 0.50 ^a^ | 0.95 ± 0.26 ^b^ |  | 0.86 ± 0.05 ^c^ | 1.58 ± 0.29 ^bc^ | 2.44 ± 0.33 ^c^ |  | 0.34 ± 0.03 ^d^ | 0.78 ± 0.09 ^b^ | 1.12 ± 0.19 ^c^ |
| KQ | 0.55 ± 0.13 ^b^ | 0.41 ± 0.16 ^a^ | 0.96 ± 0.25 ^b^ |  | 1.00 ± 0.10 ^c^ | 1.23 ± 0.23 ^c^ | 2.23 ± 0.23 ^c^ |  | 0.71 ± 0.08 ^c^ | 0.66 ± 0.12 ^b^ | 1.37 ± 0.18 ^bc^ |
| LM | 0.54 ± 0.05 ^b^ | 0.37 ± 0.21 ^a^ | 0.91 ± 0.67 ^b^ |  | 2.50 ± 0.12 ^b^ | 2.43 ± 0.14 ^ab^ | 4.93 ± 0.33 ^b^ |  | 0.96 ± 0.06 ^bc^ | 0.47 ± 0.12 ^b^ | 1.43 ± 0.12 ^bc^ |
| GV | 0.58 ± 0.08 ^b^ | 0.57 ± 0.31 ^a^ | 1.15 ± 0.38 ^b^ |  | 3.04 ± 0.05 ^b^ | 2.78 ± 0.35 ^a^ | 5.82 ± 0.40 ^b^ |  | 1.25 ± 0.13 ^b^ | 0.65 ± 0.08 ^b^ | 1.90 ± 0.20 ^b^ |
| **Pork belly** |  |  |  |  |  |  |  |  |  |  |  |
| Control | 0.50 ± 0.09 ^B^ | 0.38 ± 0.16 ^C^ | 0.88 ± 0.25 ^C^ |  | 1.93 ± 0.13 ^A^ | 1.29 ± 0.08 ^B^ | 3.22 ± 0.22 ^B^ |  | 1.80 ± 0.12 ^A^ | 0.92 ± 0.07 ^B^ | 2.72 ± 0.12 ^A^ |
| SB | 0.97 ± 0.08 ^A^ | 1.16 ± 0.06 ^A^ | 2.13 ± 0.11 ^A^ |  | 0.48 ± 0.04 ^D^ | 0.78 ± 0.08 ^C^ | 1.26 ± 0.10 ^D^ |  | 1.17 ± 0.34 ^A^ | 1.22 ± 0.08 ^AB^ | 2.39 ± 0.28 ^A^ |
| KQ | 0.23 ± 0.09 ^C^ | 0.31 ± 0.02 ^C^ | 0.54 ± 0.16 ^C^ |  | 0.53 ± 0.06 ^CD^ | 0.42 ± 0.05 ^D^ | 0.95 ± 0.12 ^D^ |  | 2.36 ± 0.15 ^A^ | 1.85 ± 0.14 ^A^ | 4.21 ± 1.64 ^A^ |
| LM | 0.61 ± 0.08 ^B^ | 0.66 ± 0.09 ^B^ | 1.27 ± 0.13 ^B^ |  | 0.68 ± 0.03 ^C^ | 1.29 ± 0.11 ^B^ | 1.97 ± 0.12 ^C^ |  | 1.22 ± 0.79 ^A^ | 1.14 ± 0.86 ^AB^ | 2.36 ± 0.38 ^A^ |
| GV | 0.85 ± 0.06 ^A^ | 0.71 ± 0.11 ^B^ | 1.56 ± 0.17 ^B^ |  | 1.41 ± 0.08 ^B^ | 2.23 ± 0.14 ^A^ | 3.64 ± 0.21 ^A^ |  | 2.24 ± 0.98 ^A^ | 1.32 ± 0.20 ^AB^ | 3.56 ± 1.18 ^A^ |

Results are presented as the mean ± standard deviation (n=3). Different lowercase letters (a-d) within the same column for pork loin or uppercase letters (A-D) for pork belly indicate significant differences (*p* < 0.05). SB, strawberry; KQ, kumquat; LM, lemon; GV, guava; CML, Nε-carboxymethyl-lysine; CEL, Nε-carboxyethyl-lysine; AGEs, advanced glycation end-product.

**Table S9.** Formulation of fruit powder barbecue sauces with and without soy sauce.

| **Ingredient** | **BBQ sauce with soy sauce (BA)** | | **BBQ sauce without soy sauce (BB)** | |
| --- | --- | --- | --- | --- |
|  | **Amount (g)** | **%Weight** | **Amount (g)** | **% Weight** |
| Fruit powder | 7.2 | 4.8 | 7.2 | 4.8 |
| White vinegar | 12.0 | 8.0 | 12 | 8.0 |
| Brown sugar | 22.5 | 15.0 | 22.5 | 15.0 |
| Soy sauce | 45.0 | 30.0 | — | — |
| Salt | — | — | 7.2 | 4.8 |
| Water | 63.3 | 42.2 | 101.1 | 67.4 |
| **Total** | **150** | **100** | **150** | **100** |
| Spice bag* | 55.5 | — | 55.5 | — |
| Black pepper* | 3.0 | — | 3.0 | — |
| Onion* | 30.0 | — | 30.0 | — |
| Garlic* | 15.0 | — | 15.0 | — |
| Ginger* | 7.5 | — | 7.5 | — |

*These ingredients were placed in a spice bag and cooked together with other ingredients. After cooking, the spice bag was removed, and only the sauce was retained.

—: Not applicable (ingredient not included in percentage calculation as it is removed after cooking).

**Table S10.** AGE extraction, LC-MS/MS conditions, and TBARS assay details.

| **Method block** | **Condition retained in supplement** |
| --- | --- |
| AGE extraction | 2 g grilled meat defatted twice with dichloromethane:n-hexane (4:1, v/v; 10 mL) by sonication for 15 min. |
| Homogenization | Residue homogenized with 12 mL deionized water. |
| Reduction | 0.45 mL homogenate mixed with 1 mL of 1 M NaBH_4_ and 0.5 mL of 0.2 M sodium borate buffer for 2 h. |
| Hydrolysis | Add 0.75 mL of 12 M HCl, centrifuge at 5,000 rpm for 5 min, then hydrolyze at 110°C for 20 h under nitrogen. |
| Internal standard step | 200 μL hydrolysate spiked with 2 μL CML-d_4_ and lyophilized; residue reconstituted in 200 μL water:acetonitrile (1:1) and filtered through a 0.22 μm nylon membrane. |
| LC-MS/MS system | Waters TQ-XS with ACQUITY UPLC. |
| LC column | BEH Amide, 2.1 × 100 mm, 1.7 μm, maintained at 60°C. |
| Mobile phase A | 20 mL/L acetonitrile, 1 mL/L formic acid, 5 mM ammonium formate. |
| Mobile phase B | 950 mL/L acetonitrile, 1 mL/L formic acid, 5 mM ammonium formate. |
| Flow rate / injection | 0.3 mL/min; injection volume 2 μL. |
| Gradient | 0-5 min 95-75% B; 5-9 min 75-65% B; 9-11 min 65-55% B; 11-11.1 min 55-30% B; 11.1-14 min 30% B; 14-14.1 min 30-95% B; 14.1-17 min 95% B. |
| MS conditions | ESI positive mode; drying gas 800 L/h at 400°C; nebulizer 7 bar; capillary 3.9 kV; MRM transitions and compound-specific settings reported separately if desired. |
| Quantification | Internal-standard calibration for CML and CEL. CML-d_4_ was used as the internal standard for quantification of both CML and CEL because isotopically labeled CEL was not commercially available. |
| TBARS sample preparation | 2 g homogenized grilled pork + 10 mL of 75 g/L TCA containing 1 g/L EDTA and 1 g/L propyl gallate; homogenize and filter. |
| TBARS color reaction | 2 mL filtrate + 2 mL of 0.02 M TBA; heat in boiling water bath for 30 min; cool and read at 532 nm. |
| TBARS calibration | 1,1,3,3-Tetraethoxypropane standard curve; results expressed as mg MDA equivalents/kg sample. |

**Table S11.** MRM transitions and compound-specific parameters for quantification of CML and CEL.

| **Compound** | **Precursor ion (m/z)** | **Product ion (m/z)** | **Dwell (s)** | **Cone (V)** | **Collision energy (V)** | **Formula/Neutral Mass** | **Retention time (min)** |
| --- | --- | --- | --- | --- | --- | --- | --- |
| CML | 205.1243 | 84.1105 | 0.230 | 4 | 24 | 204.22 | 8.75 |
| CEL | 219.2181 | 84.1087 | 0.23 | 26 | 22 | 218.25 | 8.25 |
| CML-d_4_ | 209.1543 | 88.0769 | 0.23 | 30 | 24 | 208.25 | 8.76 |

***Table S12_Polyphenol_ligand_selection is provided as a separate Excel file.***

**Table S13.** Polyphenol ligand selection, molecular docking, and MGO-trapping conditions.

| **Module** | **Operational detail retained in supplement** |
| --- | --- |
| Untargeted metabolomics | Fruit powders extracted with 80% (v/v) acetonitrile containing internal standards and analyzed on a UPLC-ESI-MS/MS platform in positive and negative ion modes. |
| Annotation rule | Metabolites annotated by MWDB matching; screening retained compounds with match score ≥ 0.7. |
| Ligand curation | Isomers consolidated, non-natural false-positive annotations removed, and candidates ranked by abundance, literature relevance, structural diversity, and structural certainty. |
| Final ligand set | 10-12 representative compounds per fruit, covering seven structural classes; docking set contained 28 polyphenols plus MGO, GO, and glucose as references. |
| Protein model | Porcine myosin-4 (MYH4, UniProt Q9TV62) structure from AlphaFold; S1 domain (residues 1-843) used as receptor. |
| Receptor preparation | Rigid receptor conversion to PDBQT using Open Babel v3.1.0. |
| Ligand preparation | 3D structures generated from SMILES with RDKit v2023.09.6 using ETKDGv3 and MMFF94 minimization (1,000 iterations), then converted with Meeko. |
| Blind docking | AutoDock Vina v1.2.5; grid box 97.5 × 117.7 × 105.2 Å centered at (9.40, -0.56, 2.65); exhaustiveness 128; num_modes 20. |
| Hotspot selection | Reference-ligand poses clustered with DBSCAN (epsilon = 8 Å, min_samples = 2); four hotspots selected based on pose convergence, binding energy, and local glycation-site density. |
| Focused docking | Polyphenols docked to the four selected hotspots for comparison with AGE precursors. |
| MGO trapping reaction | 25 μL of 2 mM polyphenol + 25 μL of 0.5 mM MGO; incubate at 37 °C for 1 h; terminate with 10 μL acetic acid. |
| MGO derivatization | Unreacted MGO derivatized with 30 mM o-phenylenediamine to form 2-methylquinoxaline. |
| MGO analysis | UPLC-PDA on BEH C18 column (2.1 × 100 mm, 1.7 μm); mobile phase 1 mL/L formic acid in water (A) and acetonitrile (B); 0-4 min 5-95% B, 4-7 min 95% B, 8.0-8.1 min 95-5% B, 8.1-12 min 5% B; flow 0.2 mL/min; injection 5 μL; detection 312 nm. |

**Table S14.** Consumer acceptance test design.

| **Item** | **Condition retained in supplement** |
| --- | --- |
| Ethics approval | Research Ethics Committee, China Medical University and Hospital, approval no. CMUH114-REC2-065. |
| Panelists | Fifty consumers aged 20-51 years recruited from the China Medical University campus. |
| Test format | Sequential monadic consumer acceptance test. |
| Scale | 9-point hedonic scale (1 = dislike extremely; 9 = like extremely). |
| Attributes | Color, aroma, saltiness, sweetness, sourness, bitterness, spiciness, overall flavor, aftertaste, and overall acceptability. |
| Order control | Sample presentation order randomized. |
| Palate cleansing | Water rinse between samples with a 2-3 min interval between tastings. |

**Table S15.** Sensory evaluation form.

**Consumer Acceptance Test**

**Page 1**

**Test Procedure:**

Samples will be presented to you one at a time. Please indicate your degree of liking for each sample. The previous sample will be collected before the next sample is presented. Please complete all questions for each sample.

The following items will be provided: 1 cup of water, 1 expectorant cup, 1 tissue, several bamboo skewers for evaluation. If you are missing any items or have any questions, please raise your hand to notify the attendant.

**Evaluation Procedure:**

1. Before starting this test, rinse your mouth with water for 30 seconds to ensure your mouth is clean and free of other tastes.
2. Open the sample and observe its color. After smelling the aroma, place the sample in your mouth and rate your degree of liking for each attribute in sequence, then proceed to other questions. Based on your most direct feeling, mark "✓" in the most appropriate position in the table below.
3. Before tasting the next sample, repeat step 1 and rest for 2-3 minutes.

**Page 2**

| **Panelist ID: _____________ Sample: ___________________** | | | | | | | | | |
| --- | --- | --- | --- | --- | --- | --- | --- | --- | --- |
| **Attribute** | **Dislike extremely** | **Dislike very much** | **Dislike slightly** | **Dislike a little** | **Neither like nor dislike** | **Like a little** | **Like slightly** | **Like very much** | **Like extremely** |
| Degree of liking for color | ☐ | ☐ | ☐ | ☐ | ☐ | ☐ | ☐ | ☐ | ☐ |
| Degree of liking for aroma | ☐ | ☐ | ☐ | ☐ | ☐ | ☐ | ☐ | ☐ | ☐ |
| Degree of liking for saltiness | ☐ | ☐ | ☐ | ☐ | ☐ | ☐ | ☐ | ☐ | ☐ |
| Degree of liking for sweetness | ☐ | ☐ | ☐ | ☐ | ☐ | ☐ | ☐ | ☐ | ☐ |
| Degree of liking for sourness | ☐ | ☐ | ☐ | ☐ | ☐ | ☐ | ☐ | ☐ | ☐ |
| Degree of liking for bitterness | ☐ | ☐ | ☐ | ☐ | ☐ | ☐ | ☐ | ☐ | ☐ |
| Degree of liking for spiciness | ☐ | ☐ | ☐ | ☐ | ☐ | ☐ | ☐ | ☐ | ☐ |
| Degree of liking for overall flavor | ☐ | ☐ | ☐ | ☐ | ☐ | ☐ | ☐ | ☐ | ☐ |
| Degree of liking for overall aftertaste | ☐ | ☐ | ☐ | ☐ | ☐ | ☐ | ☐ | ☐ | ☐ |
| Degree of liking for overall acceptability | ☐ | ☐ | ☐ | ☐ | ☐ | ☐ | ☐ | ☐ | ☐ |

**Table S16.** Sensory evaluation form for triangle test.

**Stability Test — Triangle Test Method**

**Instructions:**

This test consists of 3 samples. After evaluation, please circle the appropriate option to confirm whether these 3 samples are the same or different. Before starting the test, the following items will be provided: 1 cup of water, 1 expectorant cup, 1 tissue, 1 pair of chopsticks, etc. If you are missing any items or have any questions, please raise your hand to notify the attendant.

**Evaluation Procedure:**

1. Before starting this test, rinse your mouth with water for 30 seconds to ensure your mouth is clean and free of other tastes.
2. There are 3 samples below. Please evaluate them from left to right. After understanding the taste of each sample, select the one that tastes different from the other two.
3. Before tasting the next sample, repeat step 1.
4. If you need the attendant to add more water, clean the expectorant cup, or have other needs, or have completed this evaluation, please raise your hand to notify the attendant.

**Panelist: _____________**

**Set 1**

| **Attribute** | **862** | **954** | **614** |
| --- | --- | --- | --- |
| Appearance |  |  |  |
| Aroma |  |  |  |
| Overall flavor |  |  |  |
| Overall aftertaste |  |  |  |

**Set 2**

| **Attribute** | **282** | **537** | **786** |
| --- | --- | --- | --- |
| Appearance |  |  |  |
| Aroma |  |  |  |
| Overall flavor |  |  |  |
| Overall aftertaste |  |  |  |

*Note: Circle the sample code that is different from the other two in each attribute.*

**Table S17.** Consumer acceptance scores for grilled pork loin prepared with fruit powder-enriched BB sauces (soy sauce-free formulation).

| **Sensory attribute** | **GV** | **LM** | **SB** | **KQ** |
| --- | --- | --- | --- | --- |
| Overall liking | 6.28 ± 1.32 ^a^ | 5.44 ± 1.18 ^b^ | 6.33 ± 1.27 ^a^ | 6.14 ± 1.31 ^a^ |
| Color | 6.33 ± 1.64 ^a^ | 6.12 ± 1.74 ^a^ | 6.48 ± 1.77 ^a^ | 6.15 ± 1.24 ^a^ |
| Aroma | 5.87 ± 1.46 ^b^ | 5.76 ± 1.46 ^b^ | 6.41 ± 1.27 ^a^ | 6.48 ± 1.50 ^a^ |
| Saltiness | 5.76 ± 1.33 ^a^ | 5.00 ± 1.37 ^b^ | 5.81 ± 1.43 ^a^ | 5.89 ± 1.68 ^a^ |
| Sweetness | 6.56 ± 1.11 ^a^ | 5.48 ± 1.29 ^b^ | 6.74 ± 1.39 ^a^ | 5.81 ± 1.56 ^ab^ |
| Sourness | 6.52 ± 1.18 ^a^ | 5.52 ± 1.55 ^b^ | 6.67 ± 1.37 ^a^ | 6.14 ± 1.63 ^a^ |
| Bitterness | 6.44 ± 1.54 ^a^ | 4.44 ± 1.02 ^b^ | 6.78 ± 1.28 ^a^ | 6.12 ± 1.22 ^a^ |
| Spiciness | 6.52 ± 1.47 ^a^ | 5.73 ± 1.38 ^b^ | 6.15 ± 1.48 ^ab^ | 6.07 ± 1.71 ^ab^ |
| Overall flavor | 5.80 ± 1.18 ^a^ | 5.41 ± 1.64 ^a^ | 5.93 ± 1.43 ^a^ | 6.00 ± 1.70 ^a^ |
| Aftertaste | 5.72 ± 1.58 ^a^ | 5.68 ± 1.91 ^a^ | 6.07 ± 1.44 ^a^ | 6.19 ± 1.59 ^a^ |

Values are expressed as mean ± SD (n = 50).

Sensory attributes were evaluated using a 9-point hedonic scale (1 = dislike extremely, 5 = neither like nor dislike, 9 = like extremely).

^a–b^ Different superscript letters within the same row indicate significant differences (*p* < 0.05) by Duncan's multiple range test.

**Table S18.** Molecular docking results of 28 polyphenolic compounds and 3 AGE precursors at four glycation hotspots on porcine myosin (UniProt: Q9TV62).

| **Compound** | **Class** | **Fruit** | **MW** | **HA** | **E_hs1** | **E_hs2** | **E_hs3** | **E_hs4** | **Best Energy (kcal/mol)** | **Best HS** | **LE** |
| --- | --- | --- | --- | --- | --- | --- | --- | --- | --- | --- | --- |
| ***KQ polyphenols (n=14)*** |  |  |  |  |  |  |  |  |  |  |  |
| Hesperidin | Flavanone glycoside | KQ | 610.2 | 43 | -7.875 | -8.975 | -7.340 | -8.094 | **-8.975** | hs2 | 0.209 |
| Neohesperidin | Flavanone glycoside | KQ | 610.2 | 43 | -7.793 | -8.862 | -7.372 | -8.074 | **-8.862** | hs2 | 0.206 |
| Geranyl hydroxycoumarin | Coumarin | KQ | 298.2 | 22 | -7.104 | -7.177 | -7.739 | -6.991 | **-7.739** | hs3 | 0.352 |
| Vitexin-2″-O-rhamnoside | Flavone glycoside | KQ | 502.1 | 35 | -7.493 | -7.673 | -5.710 | -6.890 | **-7.673** | hs2 | 0.219 |
| Hesperetin | Flavanone | KQ | 302.1 | 22 | -7.232 | -7.061 | -7.468 | -7.020 | **-7.468** | hs3 | 0.339 |
| Peonidin | Anthocyanidin | KQ | 301.1 | 22 | -6.993 | -7.224 | -7.057 | -6.872 | **-7.224** | hs2 | 0.328 |
| Tetramethoxystilbene | Stilbene | KQ | 300.1 | 22 | -6.517 | -6.598 | -6.874 | -6.089 | **-6.874** | hs3 | 0.312 |
| Isosinensetin | Polymethoxyflavone | KQ | 372.1 | 27 | -6.629 | -6.839 | -6.546 | -6.451 | **-6.839** | hs2 | 0.253 |
| Tangeretin | Polymethoxyflavone | KQ | 342.1 | 25 | -6.733 | -6.666 | -6.053 | -6.379 | **-6.733** | hs1 | 0.269 |
| 5-O-Demethylnobiletin | Polymethoxyflavone | KQ | 388.1 | 28 | -6.417 | -6.665 | -6.528 | -6.348 | **-6.665** | hs2 | 0.238 |
| Nobiletin | Polymethoxyflavone | KQ | 402.1 | 29 | -6.480 | -6.419 | -5.630 | -6.105 | **-6.480** | hs1 | 0.223 |
| Fraxetin | Coumarin | KQ | 208.0 | 15 | -5.603 | -5.591 | -6.050 | -5.606 | **-6.050** | hs3 | 0.403 |
| trans-Ferulic acid | Hydroxycinnamic acid | KQ | 194.1 | 14 | -5.385 | -5.354 | -5.593 | -5.141 | **-5.593** | hs3 | 0.400 |
| Quinic acid | Cyclitol (organic acid) | KQ | 192.1 | 13 | -5.000 | -4.911 | -5.089 | -4.999 | **-5.089** | hs3 | 0.391 |
| ***Average*** |  |  |  |  |  |  |  |  | ***-7.019*** |  | ***0.296*** |
| ***SB polyphenols (n=14)*** |  |  |  |  |  |  |  |  |  |  |  |
| Procyanidin B2 | Proanthocyanidin | SB | 578.1 | 42 | -8.425 | -8.232 | -5.409 | -7.714 | **-8.425** | hs1 | 0.201 |
| Quercetin 3’-glucuronide | Flavonol glycoside | SB | 478.1 | 34 | -8.178 | -8.290 | -7.826 | -8.213 | **-8.290** | hs2 | 0.244 |
| Pelargonidin 3-glucoside (P3G) | Anthocyanin | SB | 433.1 | 31 | -8.111 | -8.042 | -6.657 | -7.411 | **-8.111** | hs1 | 0.262 |
| Cyanidin 3-glucoside (Cy3G) | Anthocyanin | SB | 449.1 | 32 | -8.023 | -8.081 | -7.167 | -7.715 | **-8.081** | hs2 | 0.253 |
| Apigenin 7-glucoside | Flavone glycoside | SB | 432.1 | 31 | -7.946 | -7.862 | -7.076 | -7.932 | **-7.946** | hs1 | 0.256 |
| Flavanomarein | Chalcone glycoside | SB | 434.1 | 31 | -7.145 | -7.916 | -6.378 | -7.528 | **-7.916** | hs2 | 0.255 |
| Dihydromyricetin | Dihydroflavonol | SB | 320.1 | 23 | -7.760 | -7.357 | -7.492 | -7.329 | **-7.760** | hs1 | 0.337 |
| Resveratroloside | Stilbene glycoside | SB | 390.1 | 28 | -7.691 | -7.733 | -6.896 | -7.529 | **-7.733** | hs2 | 0.276 |
| Ellagic acid | Ellagitannin | SB | 302.0 | 22 | -7.165 | -7.339 | -6.338 | -6.965 | **-7.339** | hs2 | 0.334 |
| Dihydrokaempferol | Dihydroflavonol | SB | 288.1 | 21 | -6.884 | -7.060 | -7.127 | -6.969 | **-7.127** | hs3 | 0.339 |
| Catechin | Flavan-3-ol | SB | 290.1 | 21 | -6.879 | -6.795 | -5.179 | -7.033 | **-7.033** | hs4 | 0.335 |
| Coumaric acid glucoside | Hydroxycinnamic acid glycoside | SB | 326.1 | 23 | -7.002 | -6.746 | -6.144 | -6.663 | **-7.002** | hs1 | 0.304 |
| Coumarin | Coumarin | SB | 146.0 | 11 | -5.212 | -4.965 | -5.920 | -5.355 | **-5.920** | hs3 | 0.538 |
| p-Coumaric acid | Hydroxycinnamic acid | SB | 164.0 | 12 | -5.552 | -4.949 | -5.261 | -5.585 | **-5.585** | hs4 | 0.465 |
| ***Average*** |  |  |  |  |  |  |  |  | ***-7.448*** |  | ***0.314*** |
| ***AGEs precursors (Reference)*** |  |  |  |  |  |  |  |  |  |  |  |
| Glucose | AGEs precursor | Ctrl | 180.2 | 12 | -4.683 | -4.795 | -5.103 | 14.300 | **-5.103** | hs3 | 0.425 |
| Methylglyoxal | AGEs precursor | Ctrl | 72.1 | 5 | -2.709 | 0.128 | -2.677 | -2.739 | **-2.739** | hs4 | 0.548 |
| Glyoxal | AGEs precursor | Ctrl | 58.0 | 4 | -2.185 | -1.910 | -2.049 | -2.077 | **-2.185** | hs1 | 0.546 |
| ***Average*** |  |  |  |  |  |  |  |  | ***-3.342*** |  |  |

*Note:* More negative binding energy values indicate stronger binding affinity. MW, molecular weight; HA, heavy atoms; LE, ligand efficiency (−ΔG/HA, kcal/mol per heavy atom); HS, hotspot. Focused docking was performed using AutoDock Vina (v1.2.5, exhaustiveness = 128) at four glycation hotspots identified by Phase 1 blind docking. Best Energy represents the strongest binding across all four hotspots. Of the 28 polyphenols, 27 (96.4%) exhibited stronger binding than glucose, the strongest AGE precursor. KQ, kumquat; SB, strawberry; Ctrl, AGE precursor control.

**Table S19.** MGO-trapping capacity of four representative polyphenols selected to represent distinct B-ring hydroxylation patterns.

| **Compound** | **B-ring pattern** | **Free OH** | **Docking ΔG (kcal/mol)** | **MGO trapping (%)** |
| --- | --- | --- | --- | --- |
| Nobiletin | All –OMe (PMF) | 0 | −6.48 | 2.1 ± 0.8 ^d^ |
| Pelargonidin-3-glucoside | Mono 4′–OH | 1 | −8.11 | 33.9 ± 1.2 ^c^ |
| Catechin | Catechol (3′,4′–diOH) | 2 | −7.03 | 65.0 ± 3.4 ^b^ |
| Dihydromyricetin | Pyrogallol (3′,4′,5′–triOH) | 3 | −7.76 | 85.2 ± 2.1 ^a^ |

^a–d^ Values are expressed as mean ± SD (n = 3). Different superscript letters indicate significant differences (*p* < 0.05) as determined by one-way ANOVA followed by Duncan’s multiple range test.

Free OH: number of free hydroxyl groups on the B-ring available for nucleophilic attack on MGO.

Docking ΔG: best binding free energy against porcine myosin (Q9TV62) glycation hotspots via AutoDock Vina.

MGO trapping assay: incubation with 0.5 mM MGO at 37°C for 1 h; residual MGO derivatized with o-phenylenediamine and quantified by HPLC-UV.

**Supplementary Figures**

CEL (*m/z* 219.22 🡪84.11)

CML-d_4_ (*m/z* 209.15 🡪88.08)

CML (*m/z* 205.12 🡪84.11)

**Fig. S1.** Chromatograms of AGEs.

**Fig. S2.** Pearson correlation heatmap between antioxidant parameters, sugar contents, antioxidant/sugar ratios, and AGE inhibition rates. **p* < 0.05, ***p* < 0.01, ****p* < 0.001.

**Fig. S3.** Accelerated storage stability (45 °C, 90 days) of SB and KQ BB (soy sauce-free) spray formulations. (A) Total color difference (ΔE) relative to Day 0. (B) Total flavonoid content (TFC) retention. (C) TBARS values of charcoal-grilled pork loin and belly treated with stored sauces. (D) AGE inhibition (%) of stored sauces applied to freshly grilled pork loin and belly. (E) Triangle test results: number of correct responses out of 10 panelists for appearance, aroma, aftertaste, and overall flavor attributes. Asterisks indicate significant discrimination from the Day 0 reference (**p* < 0.05, ***p* < 0.01, ****p* < 0.001; ISO 4120:2016). Data in panels A–D: mean ± SD (n = 3).

**Fig. S4.** Molecular docking binding energies (kcal/mol) of 28 fruit polyphenols and three AGE precursors against porcine myosin glycation hotspots. Polyphenols are grouped by source: KQ, kumquat-derived; SB, strawberry-derived; Ref, reference AGE precursors (glucose, methylglyoxal, and glyoxal). Docking was performed using AutoDock Vina 1.2.5 (exhaustiveness = 128) against four glycation-susceptible Lys/Arg hotspots on the AlphaFold-predicted porcine myosin structure (UniProt Q9TV62). Values represent the best (most negative) binding energy across all four hotspots for each compound. Complete numerical data are provided in Table S18.

**Supplementary references**

Benzie, I. F. F., & Strain, J. J. (1996). The ferric reducing ability of plasma (FRAP) as a measure of “antioxidant power”: The FRAP assay. *Analytical Biochemistry*, *239*(1), 70–76. <https://doi.org/10.1006/abio.1996.0292>

Ferreira, I. C. F. R., Baptista, P., Vilas-Boas, M., & Barros, L. (2007). Free-radical scavenging capacity and reducing power of wild edible mushrooms from northeast Portugal: Individual cap and stipe activity. *Food Chemistry*, *100*(4), 1511–1516. <https://doi.org/10.1016/j.foodchem.2005.11.043>

Molole, G. J., Gure, A., & Abdissa, N. (2022). Determination of total phenolic content and antioxidant activity of *Commiphora mollis* (Oliv.) Engl. resin. *BMC Chemistry*, *16*, 48. <https://doi.org/10.1186/s13065-022-00841-x>

Moo-Huchin, V. M., Estrada-Mota, I., Estrada-León, R., Cuevas-Glory, L., Ortiz-Vázquez, E., Vargas y Vargas, M. de L., Betancur-Ancona, D., & Sauri-Duch, E. (2014). Determination of some physicochemical characteristics, bioactive compounds and antioxidant activity of tropical fruits from Yucatan, Mexico. *Food Chemistry*, *152*, 508–515. <https://doi.org/10.1016/j.foodchem.2013.12.013>
